# Cryptic cycling by electroactive bacterioplankton in Trout Bog Lake

**DOI:** 10.1128/aem.01789-24

**Published:** 2025-06-20

**Authors:** Charles N. Olmsted, Mark Gahler, Eric Roden, Ben Peterson, James Lazarcik, Patricia Q. Tran, Maureen Berg, Donald A. Bryant, Danielle Goudeau, Rex R. Malmstrom, Mohan Qin, Katherine D. McMahon

**Affiliations:** 1 Department of Molecular and Environmental Toxicology, Universities of Wisconsin 5229 , Madison, Wisconsin, USA; 2 Trout Lake Station, Center for Limnology, University of Wisconsin-Madison 5228, Madison, Wisconsin, USA; 3 Department of Bacteriology, University of Wisconsin 205263, Madison, Wisconsin, USA; 4 Department of Geoscience, University of Wisconsin 124349, Madison, Wisconsin, USA; 5 Department of Civil and Environmental Engineering, Universities of Wisconsin 5229 , Madison, Wisconsin, USA; 6 Department of Integrative Biology, Universities of Wisconsin 5229 , Madison, Wisconsin, USA; 7 Joint Genome Institute, Lawrence Berkeley National Laboratory 229279, Berkeley, California, USA; 8 Department of Biochemistry and Molecular Biology, The Pennsylvania State University 8082, University Park, Pennsylvania, USA; University of Delaware, Lewes, Delaware, USA

**Keywords:** extracellular electron transfer, cryptic cycling, *Chlorobium*, dissolved organic matter, electroactive, electrotrophic, oxidation reduction potential, bacterioplankton, carbon emissions, ecology

## Abstract

The potential for extracellular electron transfer (EET) is a prevailing genomic feature of humic lake bacterioplankton. However, there has been little evidence for the substantial ecological contribution predicted by genetics. We hypothesized that anoxygenic phototrophic electrotrophs and accompanying heterotrophic electrogens cycle dissolved organic matter (DOM) between oxidized and reduced states. We predicted that such bacterioplankton would exhibit diel-scale oscillations due to the light dependency of photosynthesis. Using Trout Bog Lake in Wisconsin, USA, as our model ecosystem, we profiled the water column with depth-discrete metagenomic, physiochemical, and electrochemical analyses. We observed variation in oxidation reduction potential (ORP) in response to sunlight, initiating at depths populated by anoxygenic phototrophs with EET genes. We developed an automated buoy to measure electric current flow between many pairs of electrodes simultaneously, observing correlation in electron consumption to sunlight. Our results, combined with published metatranscriptomic analysis, indicate the occurrence of electron cycling between phototrophic oxidation (electrotrophic metabolism) by *Chlorobium* and anaerobic respiration (electrogenic metabolism) by *Geothrix*, involving DOM. We also repeatedly observed gradual seasonal increases in hypolimnion ORP throughout summer. These diel and seasonal patterns imply that electroactive DOM mediates the ecology of electroactive bacteria in lakes, controlling humic lake methane emissions.

**IMPORTANCE:**

We investigated the physical, chemical, and redox characteristics of a bog lake and electrodes hung therein to test the hypothesis that dissolved organic matter is being cycled between oxidized and reduced states by electroactive bacterioplankton powered by phototrophy. To do so, we performed field-based analyses on multiple timescales using both established and novel instrumentation. We paired these analyses with recently developed bioinformatics pipelines for metagenomics data to investigate genes that enable electroactive metabolism and accompanying metabolisms. Our results are consistent with our hypothesis and yet upend some of our other expectations. Our findings have implications for understanding greenhouse gas emissions from lakes, including electroactivity as an integral part of lake metabolism throughout more of the anoxic parts of lakes and for a longer portion of the summer than expected. Our results also give a sense of what electroactivity occurs at given depths and provide a strong basis for future studies.

## INTRODUCTION

Small inland lakes and especially their sediments are disproportionately important as both sources and sinks for greenhouse gases (1–4), the magnitude of which is determined by the routes of energy and carbon flux through these systems. We recently proposed that the metabolism of electroactive bacterioplankton in the water column controls emissions in unrecognized ways (5). These organisms can engage in extracellular electron transfer (EET), the process of routing electrons through the outermost membranes of cells, to perform redox reactions with colloidal or precipitated substances like iron, other metals, and quinone moieties in organic matter (6–9). Electroactive bacteria are often found in anoxic redox-transition environments such as those that occur in stratified water columns and sediments (5). However, their activity can be obscured by rapid redox cycling (i.e., “cryptic cycling”) of iron and manganese, as was found just below the oxycline in Lake Cadagno (10). In many systems, cryptic cycling may be further complicated by diverse dissolved organic matter (DOM) molecules that can serve as “electron shuttles” *via* the presence of phenolic quinone-like moieties (11).

This study builds on our prior work in Trout Bog Lake, a small, low-iron peat-bog lake with a characteristically high DOM content of roughly 20 mg C/L. The yellow-brown water color is largely due to the aromaticity of phenolic and quinoid components of this DOM, derived from lignin as well as the *Sphagnum* moss that surrounds the lake in a dense mat (12, 13). Redox-active DOM structures like quinone moieties produce a relatively high electron exchange capacity (11) in Trout Bog Lake (14). We previously hypothesized that the DOM quantity and quality of many lakes, including Trout Bog Lake, can greatly explain the observed high abundance of organisms that use reductive EET (redEET) to expel electrons, termed *electrogens*, and *electrotrophs* that use oxidative EET (oxiEET) to obtain electrons (5).

Within Trout Bog Lake, we know that numerous electroactive bacteria are present and actively transcribing the machinery to support EET (5), including a *Geothrix* sp. (an electrogen) and two distinct populations of *Chlorobium* spp. (electrotrophs). The metagenome assembled genome (MAG) of *Geothrix* sp. encodes and expresses genes for both canonical and uncharacterized redEET proteins (5). *Chlorobium* spp. are less commonly regarded as capable of EET, but both populations of these anoxygenic phototrophs encode three homologs of Cyc2, an iron-oxidizing protein (15). One homolog is transcribed at far higher levels than their oxidoreductases for other electron donors (5). One *Chlorobium* sp., referred to by Berg et al. (16) as “GSB-B,” blooms during the summer just below the oxycline, usually at ~2 m deep.

**Fig 1:**
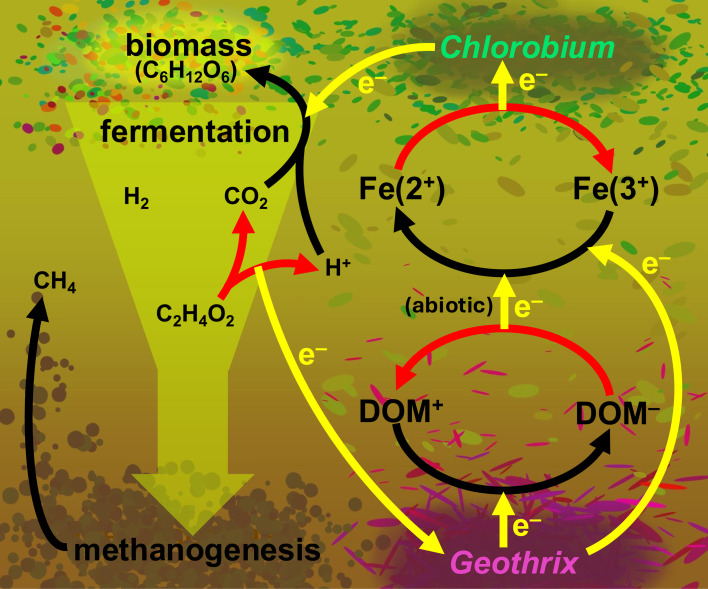
Conceptual model of electron transfer (yellow) oxidations (red) and reductions (black) from *Geothrix* to *Chlorobium*, diverting electrons from methane (CH_4_). Oxidized electroactive dissolved organic matter (DOM^+^) accepts electrons from *Geothrix* and thereby is reduced (DOM^−^). Acetic acid (C_2_H_4_O_2_) represents small organic acids or their conjugate base, acetate, formate, etc.

*Chlorobium* engaging in anoxygenic phototrophy require reducing equivalents to fix CO_2_ into biomass (17). However, less is known about their nocturnal metabolism. Some evidence exists for fermentation of glycogen stored during phototrophy, resulting in products such as acetate (18, 19). Acetate and other fermentation products are potential substrates for electrogens that would then respire and generate extracellular electron donors such as ferrous iron and reduced aromatic DOM. Based on this, we have developed a conceptual model to describe electron-shuttle-driven interactions between phototrophic electrotrophs (*Chlorobium*) and electrogens (*Geothrix*) (Fig. 1).

In the current study, we examined the vertical water column structure of Trout Bog Lake with an emphasis on redox status. We hypothesized that *Chlorobium* consume reducing equivalents during the day, creating an extracellular oxidized pool of electron shuttles, and, at night, reduced redox conditions are restored by electrogens like *Geothrix* through anaerobic respiration. Thus, each supports the other, with a net fixation of CO_2_ into microbial biomass as the zone of growth becomes denser throughout the summer. We aimed to document diurnal oscillations in redox status and seasonal changes in water column chemistry while confirming the presence and abundance of electrotrophs and electrogens using metagenomics.

## MATERIALS AND METHODS

### Trout Bog Lake characteristics

**Fig 2:**
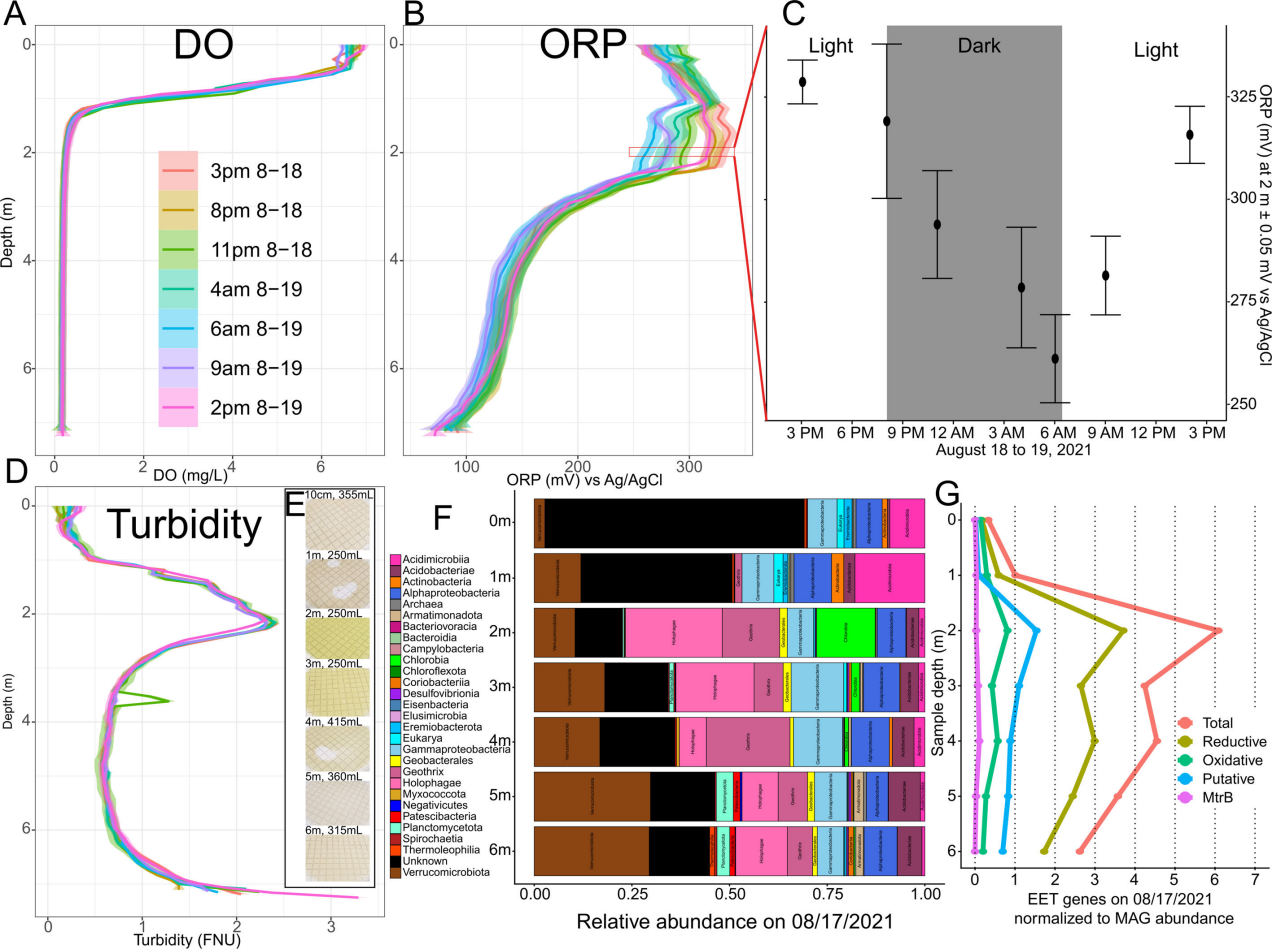
Observance of light-driven oxidation during diel sampling campaign on Trout Bog Lake including vertical profiles for (**A**) dissolved oxygen (DO), (**B**) oxidation reduction potential (ORP) vs Ag/AgCl (value +200 mV = vs SHE), (**C**) significant ORP change at 2 m deep, (**D**) turbidity, (**E**) bacterial filters, (**F**) resulting relative abundances of MAGs, and (**G**) counts of EET genes normalized to each MAG's relative abundance. Profiles were collected by slowly descending a ProDSS sonde. Each timepoint is an average of three profiles, each 20 min apart. Bacterial filters were pictured by a phone camera just prior to freezing at –80°C and later subjected to extraction and metagenomics. Normalized EET gene counts were calculated by multiplying each MAG's number of EET genes by the relative abundance of that MAG and adding all such values together per sample. Measurements and materials were collected between 17 August 2021 and 19 August 2021. ORP at 2 m error bars represent standard deviation (*N* = 20–30).

Trout Bog Lake is a darkly stained bog lake on the northwest periphery of Trout Lake, Boulder Junction, WI, and is part of the North Temperate Lakes Long-Term Ecological Research (NTL-LTER) program. This humic lake is located at the coordinates 46°02'28.2"N 89°41'10.6"W, has a surface area of 1.1 hectares, and has a maximum depth of 7.9 m. The warmer, oxygenated epilimnion (Fig. 2A) is typically only represented by the top 1–2 m throughout the summer. In full sun, only 0.008% of photosynthetically active radiation could be detected at 2 m deep and 0% by 3 m (Fig. S0). As a bog lake, its edges are covered by floating *Sphagnum* sp. moss, *Gaultheria procumbens* wintergreen, and other typical bog flora, but a small portion of the boggy edge is not far away from solid forest floor under a mix of deciduous and coniferous trees.

### Metagenomics collection and extraction

We used all Trout Bog Lake MAGs from Olmsted et al. (5), as well as additional MAGs (Table S1) generated (Text S2) from water and electrode biofilm samples (Table S2), for a total of 1,301 MAGs—597 of which were identified as bacterial genomes over 50% complete and less than 10% contaminated. Both integrated and depth-discrete water samples were collected using a peristaltic pump into an opaque Nalgene sample container and transported in a cooler with ice packs to the University of Wisconsin Trout Lake Station. Water samples were filtered (0.2 µm polyethersulfone; Pall Corporation), frozen at –20°C, and then transferred later to –80°C, except for all 2021 water samples and 2020 electrode samples which were frozen directly at –80°C. Phenol chloroform DNA extractions were performed on filters and electrodes as previously described (20).

### Genome analysis

Genome-wide average nucleotide identity (gANI) of MAGs was calculated using FastANI v1.3 (21). All MAGs were clustered under representative metagenomic operational taxonomic units (mOTUs) (Table S1) by using an identity cutoff of 98% and selecting the most complete and least contaminated MAG to represent each cluster. The displayed taxonomic abundance was derived from the reads of each sample realigned to mOTU representative sequences. Oxidative, reductive, and putative EET genes were determined using FEET (5), and the FEET pipeline definitions of redEET versus oxiEET are largely based on FeGenie (22). We used the METABOLIC V3 software (23) to determine the genetic potential for various oxidoreductases and other metabolisms on MAGs over 50% complete and less than 10% contaminated, and we only considered MAGs of the same quality when summarizing gene counts in a given sample.

### Microghost Buoy design and deployment

We designed and deployed a buoy that automatically measured electrical current flow between pairs of unpoised electrodes. The circuit board design (File. S1 MicroghostBoard.pcb), parts list, build information, and silly reasons for the name “Microghost” are available in Supplementary Text S1. We refer to the pairs of connected electrodes as “channels.” Prior to deployment, 16 pairs of 18 cm^2^ plain carbon cloth (1071 HCB; Fuel Cell Store, AvCarb) electrodes were weaved over 3 cm of exposed bundled (19 strands of 27 gauge) but otherwise insulated copper wire amounting to a copper surface area of 6.46 cm^2^ per electrode. Two buoy deployments occurred from 21 July 2019 to 13 August 2019, and the other was from 16 August 2020 to 9 September 2020. A datalogger (HOBO, Onset MX2202) was secured on top of each buoy to measure sunlight intensity. Dissolved oxygen (DO) and temperature readings are from separate buoys placed roughly 10 m away using a miniDOT Logger (Precision Measurement Engineering) (Fig. S1D through F). Some 2020 buoy electrodes (Fig. S1A through C) were covered in calcium-alginate with pores with estimated diameters of 12–16 nm (24) to test for electroactivity absent bacterial attachment. To coat electrodes, we soaked each in 1% sodium alginate, placed it into 100 mM calcium chloride to cure for one hour, and rinsed it with DI water. The same steps were repeated for a double layer. These electrodes were stored in 50 mM calcium chloride at 4°C but rinsed in deionized water before deployment.

### Chemical and biological profiling

All chemical and biological samples were collected *via* peristaltic pump and later analyzed as detailed (Text S2). Text S2 also contains statistical and additional methods. Depth-discrete cytometric counts of *Chlorobium* spp. and total cell counts were generated in 2018 by flow cytometry as previously described (16), but now, we publish cell counts rather than only percent of total cells. Turbidity, pH, chlorophyll, temperature, and DO in 2017 (Fig. S2) were collected using an Exo1 multiparameter sonde (Yellow Springs Instruments). In 2018 and beyond, the same parameters—except oxidation-reduction potential (ORP) instead of chlorophyll—were measured using a ProDSS multiparameter sonde (Yellow Springs Instruments). ORP was measured against an Ag/AgCl reference electrode. Our diel ProDSS vertical profiling procedure was as follows, all taken by a single researcher to minimize human-derived differences between profiles. All parameters were calibrated once just prior to the sampling trip. The sonde was set outside the water for 1 min and then given 5 min to equilibrate in the water at a depth of zero meters before dropping the sonde at a constant rate of roughly 1 cm every 1.5 s, logging values every 2 s. Each vertical profile took about 15 min, and thus the beginning measurement for each replicate profile was about 21 min apart. Values were logged only as the sonde was lowered.

## RESULTS

### Sunlight-driven oxidation where anoxygenic phototroph *Chlorobium* sp. is abundant

Given our prior observations (16, 25) of dense plates of anoxygenic phototrophs (primarily *Chlorobium* sp. “3m_metabat_bin_57” (Table S1) corresponding to “GSB-B”), we hypothesized that these green sulfur bacteria may alter redox potential on diel timescales because they actively express oxiEET genes (5) and require reducing equivalents during CO_2_ fixation driven by energy from sunlight. Therefore, we performed subdaily vertical profiling in 2021 on Trout Bog Lake with special attention paid to ORP, a measure of the potential between dissolved electroactive components interacting with a platinum electrode against a Ag/AgCl reference electrode. Indeed, we detected a significant (*P* < 2.2e–16) diel shifting of the ORP (Fig. 2B and C) at 2.0 m deep, just below the stable oxycline (Fig. 2A). In the region with the most diel variation of ORP, 1.0–2.5 m deep, ORP decreased gradually at night, reaching its lowest potential just before dawn. After only 2–3 h of daylight, an obvious increase in ORP (oxidation occurring) initiated at 2.0 m, the depth the genus *Chlorobium* was found to be most abundant (Fig. 2E and F). Later in the day, the ORP of the entire region returned to more oxidized values as observed prior to dusk the previous day (Fig. 2B and C).

### Unexpected seasonal oxidation in Trout Bog Lake hypolimnion

**Fig 3:**
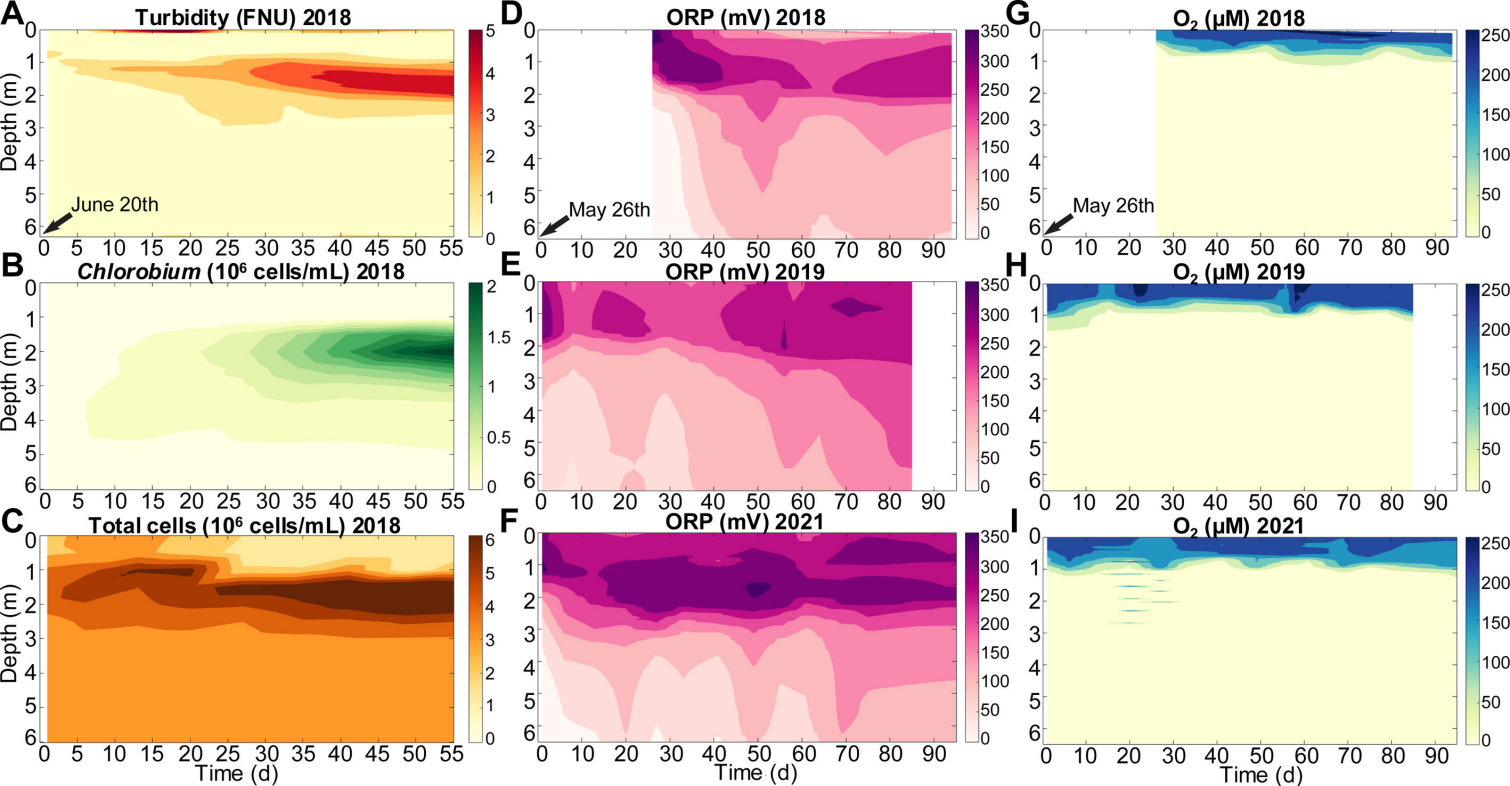
Seasonal measurements from Trout Bog Lake including (**A**) turbidity, (**B**) cytometric counts of *Chlorobium* and (**C**) total cells in 2018, (D through F) oxidation reduction potential (ORP) vs Ag/AgCl (value + 200 mV = vs SHE), and (G through I) dissolved oxygen for 2018, 2019, and 2021 summers. The beginning date for each column of graphs is indicated, and a blank space is added so the date for each year is aligned vertically. Cytometric data were collected by peristaltic pump. All other data were collected by slowly dropping down a ProDSS sonde.

We collected weekly vertical profiles of standard limnological measurements with a multimeter sonde over between May and August, as part of our routine sampling efforts through the North Temperate Lakes Microbial Observatory. Upon examination of ORP, we were surprised to notice a seasonal change in potential (Fig. 3; Fig. S3). Since ORP is a value that depends on the ratios and electroactive tendencies of reductants and oxidants, including DOM (26–28), we use ORP as a proxy for the overall redox status of the solute pool in the lake. We further interpret the value as primarily reflecting the redox state of DOM because the electron exchange capacity of DOM in Trout Bog Lake is at least one order of magnitude higher than each other measured chemical that likely also contributes to the ORP, such as iron and sulfide (14) (Table S3; Fig. S4).

Fundamental environmental microbiology concepts would predict that, in a strongly stratified, anoxic body of water, the redox status would gradually decrease from the onset of spring stratification until fall mixing. Diverse bacteria will respire terminal electron acceptors roughly in sequential order (29) down the redox ladder (30), creating highly reduced conditions in the hypolimnion. However, we observed the opposite. The ORP of the hypolimnion achieved its most reduced state within weeks after spring mixing and thereafter trended toward becoming more oxidized. We observed this for the summers of 2018, 2019, and 2021 (Fig. 3D through F). Summer of 2020 had too few sample dates to detect trends due to pandemic-imposed fieldwork restrictions. From our earliest spring profile to the latest in August 2018, 2019, and 2021, the average ORP from 2–6 m respectively increased by 166 mV, 110 mV, and 128 mV.

### Water column and electrode biofilm microbial communities

We collected flow cytometric data (16) and sequenced metagenomes from routine depth-integrated water column samples and from additional depth-discrete water samples collected in 2021 (Fig. 2E; Table S2). This sampling yielded MAGs similar to those found by previous depth-integrated sampling of Trout Bog Lake (5, 31) (Fig. 2F; Fig. S5; Table S1), with some exceptions (Text S2). Generally, MAGs recovered were affiliated with common freshwater taxa (32). Notably, many of these MAGs harbored genes involved in either oxiEET, redEET, and sometimes both in the same MAG (Table 1; Table S1). The most abundant bacterial family found at 2 m and below was *Holophagaceae*, the most common genus of which overall was the electrogen *Geothrix*. The next most abundant bacterial genus was *Chlorobium*, found maximally at 2 m.

**TABLE 1:** Highlighting Trout Bog Lake bacteria with EET genes

Bacterium	Taxonomic level	Proportion EET + MAGs	redEET genes	oxiEET genes	Relevant depth (m)
*Chlorobium*	Genus	1	0	3	2–3
*Geobacter*	Genus	1	31–47	1–2	2–6+
*Geothrix*	Genus	1	7–10	0	1–6+
*Holophaga*	Genus	1	1–10	0–1	2–6+
*Novosphingobium*	Genus	0.86	0	0–2	0–1
*Polynucleobacter*	Genus	0.43	0–1	0–3	0–3
*Rhodoferax*	Genus	1	0–5	3–6	4
*Terracidiphilus*	Genus	1	3–8	0–3	2–5
*Acetobacteraceae*	Family	0.86	0–3	0–5	0–6+
*Ferrovaceae*	Family	1	3–8	0–3	1–5
*Magnetospirillaceae*	Family	0.92	0–1	0–8	2–4
*Steroidobacteraceae*	Family	0.90	0–4	0–2	0–1, 4–5
Actinobacteria	Phylum	0.03	0–8	0–3	0–1
Verrucomicrobiota	Phylum	0.83	0–9	0–2	0–6+

**Fig 4:**
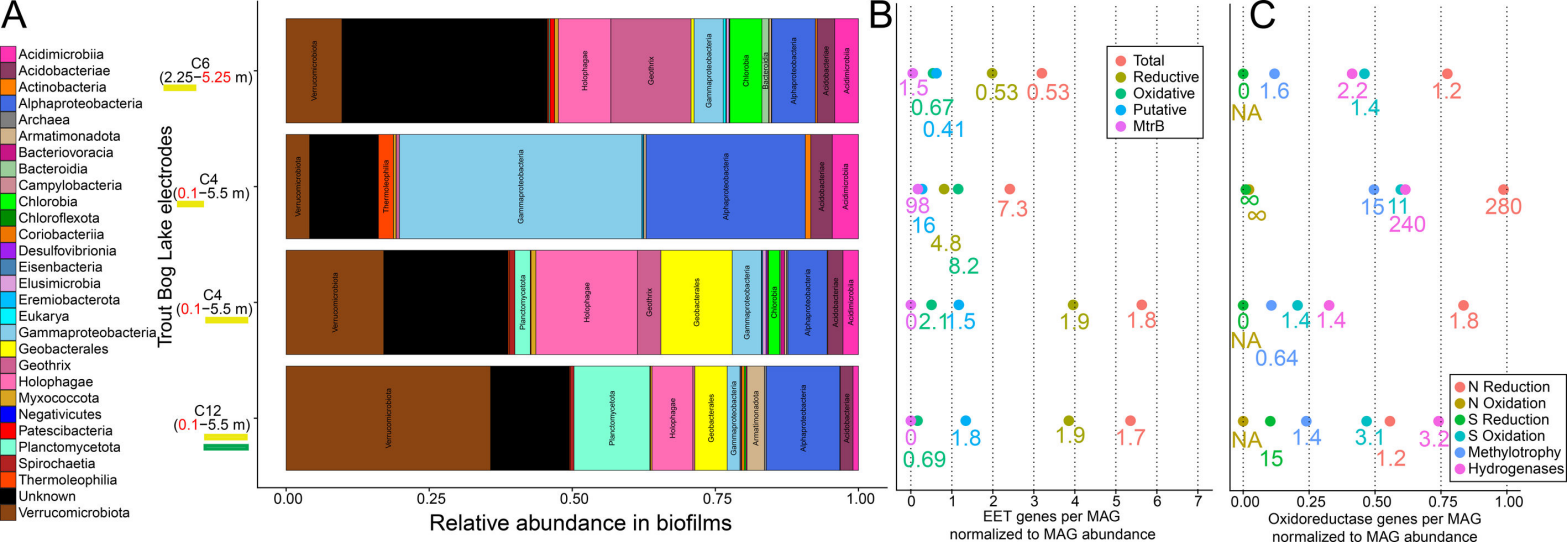
Microghost Buoy electrode biofilm MAG (**A**) taxonomy, (**B**) EET genes, and (**C**) other oxidoreductase genes normalized to the relative abundance of MAGs. Each row represents data from one electrode in a pair of electrodes connected by 4.75 Ω, a “channel,” and the depth of the represented electrode has a yellow underline. The green underline indicates the electrode covered in porous calcium alginate. The channel number above the anode (black text) and cathode (red text) depths corresponds to the 2020 channel numbers in Fig. S1. Normalized gene counts were calculated by multiplying each MAG's number of the given gene type by the relative abundance of that MAG and adding all such values together per sample. Relative abundance represents the average metagenomic read mapping for all mOTUs in the given clade, and displayed subclades were not double counted in higher clades. The point labels represent the point's value divided by the value found for water samples near the same depth, 2 m water sample for the 2.25 m electrode, and the average of 5 m and 6 m water samples for the 5.5 mdeep electrode. NA represents when the value was zero for both while infinity symbols are for when only the water sample value is zero.

Metagenomes were also recovered from four electrodes after a 17-day incubation on the 2020 Microghost Buoy (Fig. 4). Most of the diversity in electrode biofilms consisted of what we define as electrode microbes; that is, any MAG for which its mOTU (98% gANI) was recovered at the highest quality from an electrode rather than a water sample. Notably, the most dominant electrogen of the water column, *Geothrix* sp., was an electrode microbe by that definition. Also, among the most abundant members in anodic biofilms were EET + water-column microbes. Electrode biofilms were also enriched with bacteria containing EET genes. Specifically, the number of EET genes normalized by the relative abundance of each MAG was higher on electrodes than in the water, except for the water at 2 m populated largely by *Geothrix* and *Chlorobium* (Fig. 4B). The cathode had more oxiEET than redEET genes, while the three anodes had far more redEET than oxiEET genes, by both metrics of diversity (Table S1) and relative abundance (Fig. 4B). *Geobacter* sp., an electrogen with numerous redEET genes, was also enriched on the anodes at 5.5 m compared to surrounding water (Fig. 2F and 4A).

### Influence of sunlight on electrical current between *in situ* electrode pairs

**Fig 5:**
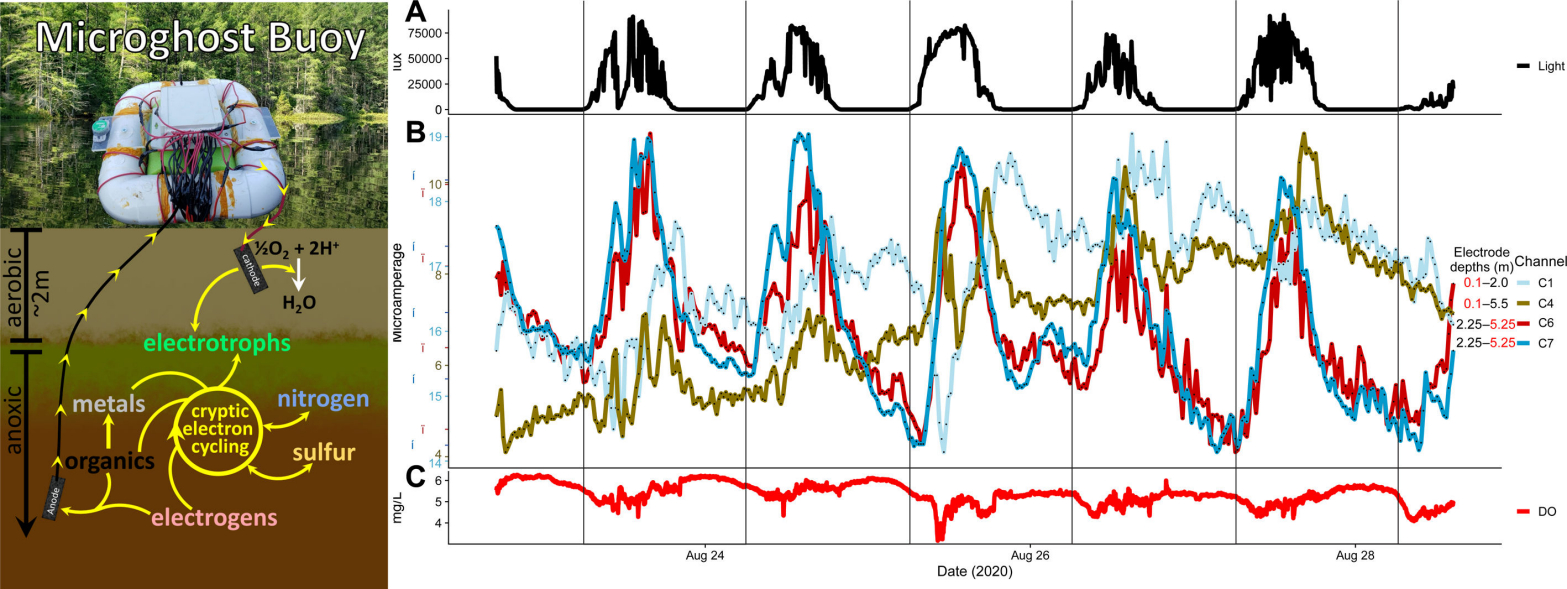
Concept figure of Microghost Buoy function (Left) and time series data from 2020 buoy deployment including (**A**) surface light intensity, (**B**) electrical current measurements for all channels, and (**C**) dissolved oxygen (DO) measured at 20 cm deep. Direction of electron transfer across 4.75 Ω is indicated by yellow arrows. The background picture is of Trout Bog Lake, and a picture of the Microghost Buoy during its 2020 deployment is pasted on top. Depths of unpoised cathodes (red text) and anodes (black text) is as indicated. Negative microamperage indicates that the direction of current flow was opposite of what we anticipated during setup, but the device was built to accommodate −50 to +50 μA. All electrodes were plain carbon cloth connected by an exposed portion of otherwise insulated copper wire. For all electrodes deeper than 10 cm, the wires were bundled together, and the electrodes were splayed out horizontally from the main bundle as to not risk touching each other. All 10 cm deep cathodes were fixed in place under the buoy's shadow.

To detect electrical variation that we suspected might occur given sufficient diurnal phototrophic electrotroph activity, we deployed an automated buoy (Fig. 5) that measured the microamperage between pairs of unpoised carbon cloth electrodes connected by a low (4.75 Ω) resistance. We deployed this buoy alongside other automated buoys measuring physical variables. There was a general increase in electron flow over the course of the time series (Fig. S6 and S7), which is consistent with the growth of EET-capable bacterial biofilms. For channels with a deep (5 ± 1 m) electrode in anaerobic water and a shallow electrode in oxygen-rich water, electrical current was as expected: overall positive as electrons flowed from the deep anode toward the shallow cathode. A more detailed explanation of observed electrical patterns is provided (Text S2), but generally, sunlight intensity significantly correlated to decreased electron transfer from all anodes located in *Chlorobium*-abundant depths (2.5 ± 0.5 m) to corresponding cathodes more so than for the channels with deeper anodes.

To test for diel variations in the *Chlorobium*-abundant zone absent the impacts of near-surface dynamics, we connected some channels (Fig. S1A, B, C6, C7, and C10) from 2.5 ± 0.5 m to 5 ± 1 m, where we did not expect as much diel variation. These channels (including Channels 6 and 7 in Fig. 5B) show negative current to indicate that the electrodes that acted as the anode or cathode were opposite of what we anticipated—i.e., the direction of electron flow was from 2.5 ± 0.5 m toward 5 ± 1 m rather than the reverse. For these channels, unlike other channels, positive correlations to the diel component of current correspond to decreased electron flow away from 2.5 ± 0.5 m, and therefore, their generally positive correlation to light is consistent with the activity of electrotrophic *Chlorobium*. Furthermore, by multivariate linear regression modeling, light was the best predictor of decreases in electron flow from anodes over other major metabolism-modulating factors, temperature and DO, but only for modeled channels with an anode in the *Chlorobium*-abundant zone (Table S4).

## DISCUSSION

### Cryptic electron cycling by anoxygenic phototrophs and electrogens

Building on previous metagenomic and metatranscriptomic analyses (5), this manuscript provides additional lines of evidence for EET-based electron cycling between *Chlorobium* sp. and electrogens in the upper layers of the Trout Bog Lake hypolimnion including EET gene enrichment in bacteria at *Chlorobium*-abundant depths (Fig. 2E through G), correlations in electrical current production with sunlight, and, most convincingly, the diel fluctuation in ORP. Also, the mere maintenance of such high ORP below the oxycline and in the domain of reductive heterotrophs (33) points to oxiEET (Fig. 3).

Given the abundance of DOM as an electroactive species in Trout Bog water (14) (Table S3), oxiEET entails the oxidation of reduced quinone moieties. We have not yet determined *via* assays that would require isolated *Chlorobium* sp. whether DOM is a direct or an indirect electron donor to the *Chlorobium* sp. oxiEET proteins. However, reduced DOM (e.g., AQDS) can quickly reduce oxidized iron (8, 34); thus, even if ferrous iron is the dominant substrate transferring electrons to the oxiEET proteins, coupled redox cycling of DOM and iron could sustain and amplify *in situ* cryptic electron cycling between electrotrophic *Chlorobium* sp. and electrogens such as *Geothrix* sp. (Fig. 1). Considering this, and taking into account potential redox chemistry (Table S3; Fig. S8) with the platinum ORP electrode (26–28), the net redox difference recorded by ORP (Fig. 2B) likely does not fully capture the gross rate of electron flux due to *Chlorobium* sp. metabolism. This is consistent with the comparatively high co-occurrence of gene expression for oxiEET, redEET (5), photosynthesis, and respiration (35) in Trout Bog Lake.

The potential for EET-driven electron cycling was greatest at 2 m and decreased with depth in the hypolimnion, based on the average number of EET genes per MAG normalized by MAG relative abundance (Fig. 2G). Without normalizing by abundance, EET genes per MAG increased below 2 m (Fig. S9A). This supports our conceptual model describing how EET operates in Trout Bog Lake: at the intersection of the light and DO gradients, anoxygenic phototrophic electrotrophs, particularly *Chlorobium* sp. GSB-B, proliferate alongside electrogens like *Geothrix* sp., providing primary productivity-derived organic compounds, oxidized iron, and presumably oxidized DOM (Fig. 1). As generated extracellular electron acceptors (e.g., oxidized quinone moieties) and labile organic compounds become scarcer deeper in the water (further away from *Chlorobium* sp.), bacteria that rely on EET-independent metabolisms (Fig. S9D) become more prevalent. Meanwhile, bacteria that still rely on EET may require more EET gene redundancy, and therefore expression, to achieve a higher concentration and diversity of EET membrane proteins to access the increasingly scarce yet diverse EET substrates.

The electrical current waveforms of Microghost Buoy channels in conjunction with the overall increased current flow over time as biofilms grew (Fig. S6 and S7) may relate to the cryptic cycling of electroactive substrates and EET metabolism by acting as a sensor for the diel activity of *Chlorobium* sp. We expected that if we connected an electrode in anoxic, reduced bog water to another electrode near the surface in more oxidized, oxygenated water, electrons would flow upwards as both reduced DOM and electrogens in the anoxic region donate electrons to the electrode (Fig. 5), similar to the microbial snorkel concept (36). Overall, this was the case, even for channels connected from the oxygenated water to the even higher-ORP yet anoxic *Chlorobium*-abundant depths (~2 m). This result suggests that the electrode biofilms contributed more to current generation than reduced molecules in the surrounding water interacting directly with the electrode surface.

Several factors clearly influenced electrical currents, but the decreases in total electron donation to electrodes from biofilms in the *Chlorobium*-abundant zone were most strongly determined by sunlight and, so this suggests, *Chlorobium* sp. GSB-B “photoelectrotrophy,” whether directly or mediated by the resulting oxidized DOM and iron. This is exemplified by our multivariate analysis (Table S4) and how channels' electrical patterns follow increases and decreases in sunlight (Fig. 5). We note how channels with an aerobic cathode subject to surface variability and an anode in the range of *Chlorobium* sp., like Channel 1, typically decreased shortly after sunlight became available for photoelectrotrophy (Fig. 5). This pattern is even more apparent for channels that were affected only by variation within the range of *Chlorobium* sp. and below, including Channels 6 and 7 (Fig. 5): whenever sunlight increased, within minutes, the current began shifting toward zero, and whenever clouds obscured the sun, this reversed as electrons otherwise occupied by photoelectrotrophy were permitted to donate to the anode.

Meanwhile, the fluctuations of Channel 4 likely represented electrochemistry on its aerobic cathode, considering the expectedly steady donations from electrogens and reductive chemistry to its deep anode (Fig. 5). The influence of possible electroactivity on aerobic cathodes was considered, particularly as biofilms grew, likely providing microenvironments for both anaerobic electrogens and microaerophilic electrotrophs. Light-dependent electrogenesis has been shown in some organisms (37) including some cyanobacteria to compensate for overcharged photosystems (38). This idea is supported by strong negative correlations with sunlight for cathodes at 70 cm (Fig. S1A, B, C5, and C13) where one expects more aerobic phototrophs based on 2017 chlorophyll data (Fig. S2).

We observed the consistent and repeatable phenomenon of current flow opposite to the initially anticipated direction from evidently anodic electrodes in *Chlorobium*-abundant depths (2.5 ± 0.5 m) to cathodic electrodes at more reduced, deeper depths (5 ± 1 m). This suggests there was something—whether biotic or abiotic—around 5 ± 1 m and/or on the electrodes in that depth range capable of accepting electrons at the prevailing redox potential maintained by the shallower electrode (Fig. 5B; Fig. S1A, B, C6, C7 and C10). Unfortunately, the electrode from which we attempted to sequence biofilm DNA to see if these deep cathodes were enriched for electrotrophs yielded abnormal traces and thus had to be excluded from shared-lane sequencing.

However, our available pelagic metagenomes yielded potential explanations: The greater abundance of electrogens at 2 m, indicated by increased turbidity and MAG relative abundance, explains why their electrogenic respiration might overpower the electrogenic respiration at deeper depths. Furthermore, the electron acceptance from electrodes at 5 ± 1 m is consistent with the uptick of oxiEET at 4 m (Fig. 2G; Fig. S9A) due to *Rhodoferax*, *Ferrovaceae*, *Magnetospirillaceae*, and *Acetobacteraceae* (Table S1). Given the potential for chemoautotrophy in these organisms, the substrate availability at 5 ± 1 m, and present hydrogenases (Fig. S9C and D), the terminal electron acceptor may be CO_2_. The presence of sulfur and nitrogen-compound oxidoreductases (Fig. S9B) suggests that these substrates might also be used, but neither sulfate nor nitrate is measurable at this depth.

### Influence of electrotrophic *Chlorobium* spp. oxidation on nitrogen and sulfur cryptic cycles

Diverse sulfur and nitrogen-cycling microbes capable of EET that may be sensitive to changes in the overall redox state would be influenced by the oxidative activity of *Chlorobium* spp. 2 m and below. *Chlorobium* spp. may also influence them more directly through ecological interactions like competing for reduced substrates or providing reducible and consumable substrates. Notably, we observed slight yet significant (*P* < 2.2e–16) diel variability in ORP between 3.5 and 6 m (Fig. 2B), and, while DOM is likely the dominant electroactive component, ORP is a summary measurement of many electroactive substrates including sulfur, nitrogen, and other compounds (Fig. S4).

There may be multiple explanations for why we observed diel variability in ORP below 3.5 m, such as minor levels of *Chlorobium* spp. oxiEET and/or sulfur oxidation. There is a low yet measurable count of *Chlorobium* spp. in this range (Fig. 3B), potentially photosynthesizing in very dim light (39), considering that our photometer was not sensitive enough to detect trace scattered red and infrared light (Fig. S0). Closer to the sediment, sulfide contributes more to decrease the ORP, but there was a slight peak of sulfide at 3 m (Fig. S4B), suggesting that there may have been some sulfur cycling, potentially by *Chlorobium* spp. and other organisms. Also, the concurrence of oxidoreductases and EET genes, and their abundance at particular depths (Fig. S9), raises the possibility that EET could have facilitated cryptic electron cycling between various redox states of sulfur, nitrogen [e.g., references (7) and (40)], iron, and DOM. Based on abundance at 4 m and their genes, we identified multiple bacteria that might play a role in this EET-involved cryptic redox cycling (Table 1; Text S2).

### Oxidative EET controls summertime ecology in Trout Bog Lake, obstructing methanogens

Competition for simple organic acids resulting from the use of oxidized DOM as an electron acceptor for anaerobic respiration has been suggested to be a controlling factor for methanogenesis in humic-rich systems (12, 33, 41, 42). Lau et al. (33) outlined this interaction regarding oxygen-linked redox cycling of DOM with anaerobic respiration near the oxycline. Our results indicate that photoelectrotroph-linked DOM oxidation could promote such respiration entirely within the anoxic hypolimnion.

In addition to *Chlorobium spp*., there is a diversity of putatively electrotrophic bacteria (5) (Table S1) that may be responsible for the hypolimnion ORP gradually increasing throughout each summer, especially considering that this seasonal increase extends well below where *Chlorobium* sp. (GSB-B) was the most abundant (Fig. 2 and 3). Alternatively, recent advances have shown that DOM-related electron hopping can extend over longer distances than previously considered (43). Regardless of which electrotrophic organisms and abiotic phenomena were responsible, the key point is that the balance between oxidative and reductive EET-driven electron cycling shifted over the summer. In turn, the resulting increase in abundance of oxidized compounds as electron acceptors for anaerobic respiration would be expected to suppress methanogenesis (44).

Another way that hypolimnetic EET-driven electrotrophy could suppress water column methanogenesis is by simply providing a large quantity of anoxygenic photosynthetic and dependent electrogenic planktonic biomass with a means to remain alive until fall mixing. Particularly in the case of a partial fall mix as observed at least sometimes (if not usually) in Trout Bog (45), all the living biomass which does not settle out beforehand will be exposed to oxygen, permitting more zooplankton grazing (46), aerobic respiration, and oxygen-requiring degradative pathways. This may also be true for a full mix, but most fermentable biomass produced by primary production would be above to just below the turbidity peak (Fig. 3), and exposure to oxygen-dependent mineralization might make some deeper carbohydrates fermentable (33, 47). Hypothetically, in the absence of either cryptic EET cycling or the overall hypolimnetic oxidation, the settling biomass from primary production would be not only richer in easily fermentable substrates but also on a more direct route—i.e., not suspended all summer long—to the zones occupied by methanogens, thereby providing fermenters and *ipso facto* colocalized methanogens with a steady, labile feedstock.

### Conclusion

By profiling the water column of a darkly stained lake with depth-discrete metagenomic, physiochemical, and electrical analyses, we have provided evidence in alignment with previous findings that anoxygenic photoautotrophs in such lakes can generate volumes of oxidized DOM *via* electrotrophy that can be re-respired by electrogens. This cryptic cycling and likely additional oxiEET may seasonally cause gradual oxidation of DOM throughout much of the hypolimnion. Considering the broad distribution of this particular blooming *Chlorobium* sp. and the global prevalence of anoxygenic phototrophs carrying oxiEET genes (5, 25), our extended knowledge of this phenomenon has widespread implications for ecosystem-scale methanogenesis repression and bacterial ecology.

We observed very clear diurnal oxidation in response to light initiating from the depth at which the dominant phototrophic electrotroph (*Chlorobium* sp.) in Trout Bog Lake is the most abundant. Comparing the concentration and electron exchange capacity of DOM to that of inorganic chemicals in Trout Bog Lake implies that electroactive DOM contributes in a crucial way to the metabolism of oxiEET-capable anoxygenic phototrophs and dependent electrogens.

Our findings imply that even though organic matter is the main carbon and energy source that eventually feeds methanogenic activity, higher proportions of electroactive DOM, such as molecules with quinone moieties produced by *Sphagnum* or microbially derived mediators, can produce unfavorable conditions for methanogens in anoxic hypolimnia because electroactive DOM is a regenerable substrate powering their competitors. Electroactive DOM is not just regenerable by oxygen and mixing events, but it is also re-oxidized by diverse anaerobic microbes, in particular *Chlorobium* spp. and other anoxygenic phototrophs. Such processes contribute to the oxidation of materials trapped in the hypolimnion, providing an expansive anaerobic environment replete with electron acceptors for diverse electrogens to respire while consuming fermentation products that could otherwise be consumed by methanogens.

## Data Availability

Sequencing data are published under the NCBI Bioproject ID PRJNA1018295. Metagenome assembled genomes are published at https://osf.io/kmj2s/.

## SUPPLEMENTAL MATERIAL

Text S1 aem.01789-24-s0001.pdf Microghost Buoy design details.

Text S2 aem.01789-24-s0002.pdf Additional methods and text.

Supplemental figures aem.01789-24-s0003.pdf Figures S0 to S10.

Supplemental legends aem.01789-24-s0004.pdf Legends for Tables S1 to S5.

Table S1 aem.01789-24-s0005.csv Metagenome assembled genome (MAG)-specific information.

Table S2 aem.01789-24-s0006.csv Sample-specific information.

Table S3 aem.01789-24-s0007.xlsx Electroactive chemical species in Trout Bog Lake.

Table S4 aem.01789-24-s0008.xlsx Multivariate linear regression analysis summary with diel component of current as the dependent variable.

Table S5 aem.01789-24-s0009.xlsx Key of abbreviations.

ASM does not own the copyrights to Supplemental Material that may be linked to, or accessed through, an article. The authors have granted ASM a non-exclusive, world-wide license to publish the Supplemental Material files. Please contact the corresponding author directly for reuse.
