# Supplementary material for: Cryptic cycling by electroactive bacterioplankton in Trout Bog Lake
Source: Appl Environ Microbiol. 2025 Jun 20;91(7):e01789-24. doi: 10.1128/aem.01789-24 (PMC12285243; doi:10.1128/aem.01789-24)
Supplement: Text S1 — Microghost Buoy design details. [file aem.01789-24-s0001.pdf]

Supplementary Text S1.

The Microghost Buoy

The Microghost Buoy was named as a pun on supernatural cinema where they measure "ghosts" using "EMF," electromagnetic force—or fields depending on the show or movie. Either way, the Microghost Buoy indirectly measures EM force, the cause of voltage, or EM fields; that is, fields related to moving electric charges. The Microghost Buoy was designed to track electrical current flow through a set of 16 channels set to a range of (-50 to +50 µA) by measuring potential difference (voltage) across a 4.75 Ω resistor at regular intervals (V=IR). We used this device to assess microbe-related patterns in electrical currents as microbial biofilms grew on electrodes attached to the ends of each channel. Or, if cinema is to be believed, perhaps we were measuring the ghosts of microbes.

Microghost Buoy circuit board parts list

DIGIKEY.COM

Part	Digikey #	Quantity
Op-amp MAX4239 SOIC8	MAX4239ASA+-ND	16
Op-amp TLE2426IDR SOIC8	296-1345-1-ND	16
Resistor, Shunt 0.1% 4,75	A105751-ND	16
Resistor, gain R2 470K	RG32P470KBCT-ND	16
Resister, gain R1 220	P220BCCT-ND	16
Feather Basic Proto	1528-1514-ND	
Adalogger	1528-1621-ND	
Header kit	1528-1581-ND	
SD card		
Battery		
Timer	1528-2379-ND	
Terminal blocks 8-wire	277-1279-ND	4
Silicone conformal coating	473-1354-ND	

MOUSER.COM

Part	Mouser #	
MCP3428-E/SL	579-MCP3428E/SL	4
Bypass caps 0.1uF	710-885012208009	5
Header, RA	538-90121-0124	1

POLOLU.COM

Part	Pololu #	
Voltage Regulator U1V11F5	2562	1

Microghost circuit board setup

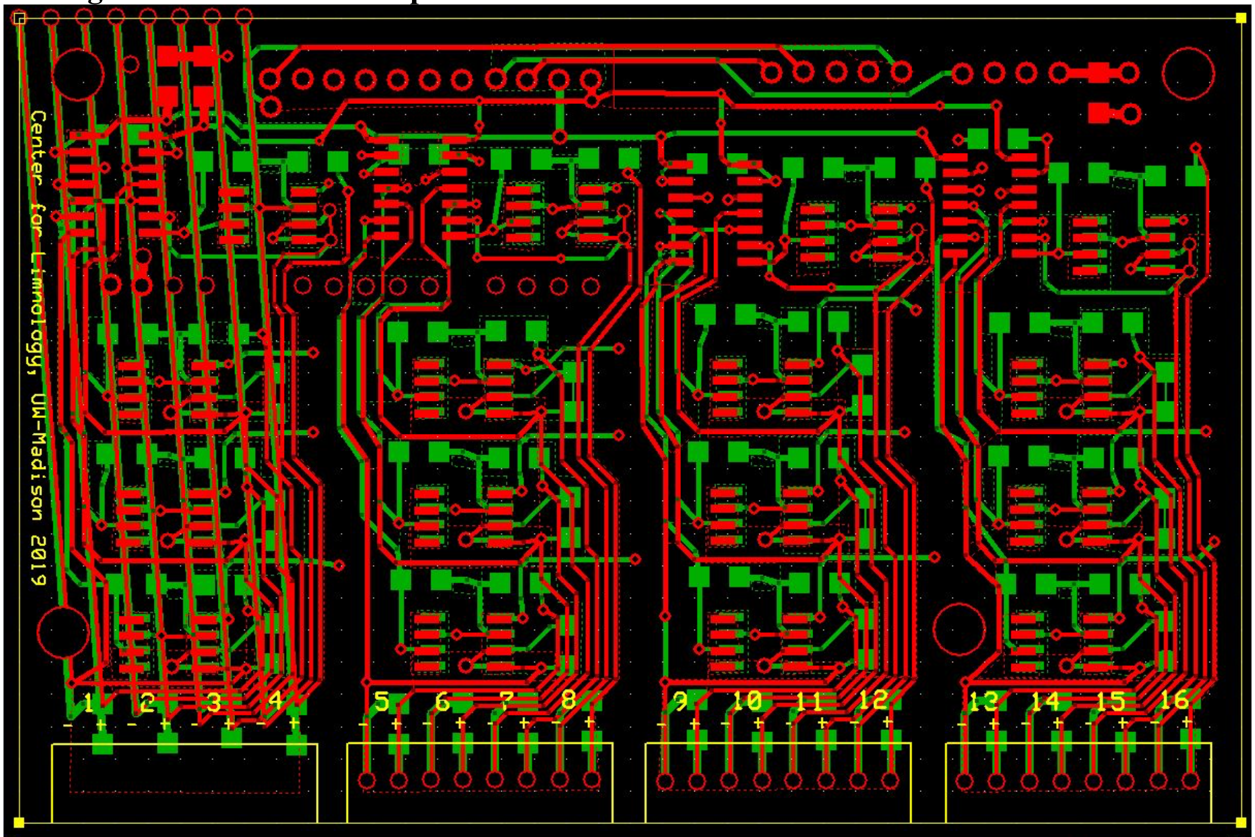

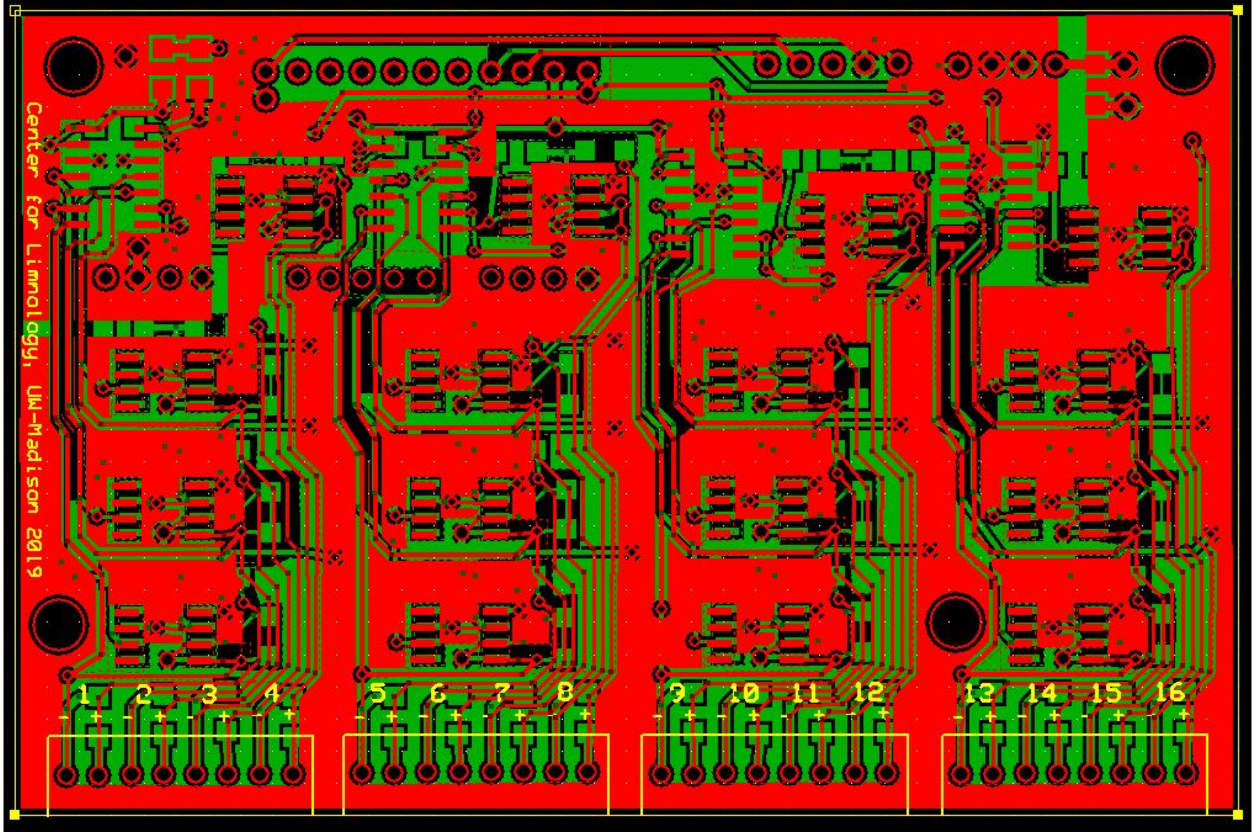

Partially built	final product
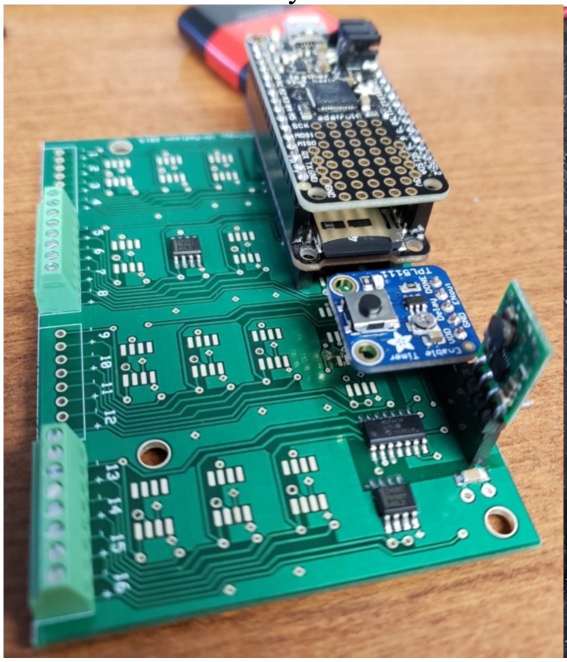	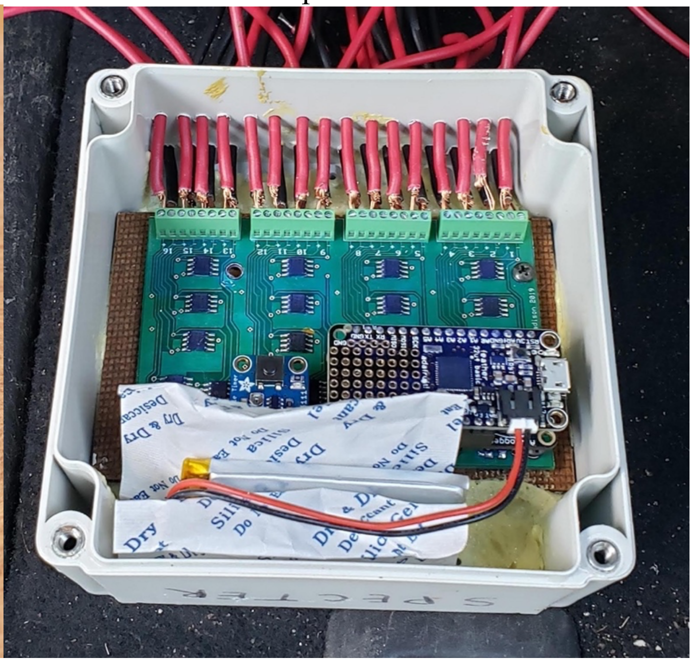

Microghost Buoy result manipulations:

Converting device voltage output (V) to microamperage (A) representative to what was flowing across 4.75Ω resistors:

A = (V*(10^6)) / 40590 (in µA)

Corrective equation applied to current (A) to convert to a slightly more accurate reading compared to a variable direct current power supply (A2):

A2 = 0.0212*A - 0.2268 (in µA)

Microghost Buoy R selector:

i_max/min	R shunt	Vin	R1	R2	Amp_Gain	Vo_1	PGA	Vo_2
0.00005	4.75	0.000238	220	470000	2136.363636	0.507386	4	2.029545

Common R 10,12,15,18,22,27,33,39,47,56,68,82

Microghost Buoy power budget:

	microAmps	N/board	milliAmps	samps/hour	samps/1Ahr
Op-amp MAX4239	600	16	9.6		
Op-amp TLE2426	170	16	2.72		
ADC - Microchip	135	4	0.54		
Feather		1			
Adalogger		1			
5V converter		1	2		
			14.86	14400	969.0444

sample periods per day: 48

Microghost Buoy time setting example Arduino code (run first):

```
// Date and time functions using a DS1307 RTC connected via I2C and Wire lib
```

```
#include <Wire.h>
#include "RTClib.h"

RTC_PCF8523 rtc;

char daysOfTheWeek[7][12] = {"Sunday", "Monday", "Tuesday", "Wednesday", "Thursday", "Friday", "Saturday"};

void setup () {

  while (!Serial) {
    delay(1);  // for Leonardo/Micro/Zero
  }

  Serial.begin(57600);
  if (! rtc.begin()) {
    Serial.println("Couldn't find RTC");
    while (1);
  }

rtc.adjust(DateTime(F(__DATE__), F(__TIME__)));
/*
  if (! rtc.initialized()) {
    Serial.println("RTC is NOT running!");
    // following line sets the RTC to the date & time this sketch was compiled
    rtc.adjust(DateTime(F(__DATE__), F(__TIME__)));
    // This line sets the RTC with an explicit date & time, for example to set
    // January 21, 2014 at 3am you would call:
    //rtc.adjust(DateTime(2019, 4, 10, 12, 5, 0));
  }
*/
}

void loop () {
    DateTime now = rtc.now();

    Serial.print(now.year(), DEC);
    Serial.print('/');
    Serial.print(now.month(), DEC);
    Serial.print('/');
    Serial.print(now.day(), DEC);
    Serial.print(" (");
    Serial.print(daysOfTheWeek[now.dayOfTheWeek()]);
    Serial.print(") ");
    Serial.print(now.hour(), DEC);
    Serial.print(':');
    Serial.print(now.minute(), DEC);
    Serial.print(':');
    Serial.print(now.second(), DEC);
    Serial.println();

    Serial.print(" since midnight 1/1/1970 = ");
    Serial.print(now.unixtime());
    Serial.print("s = ");
    Serial.print(now.unixtime() / 86400L);
    Serial.println("d");



    Serial.println();
    delay(3000);
}
```

Microghost Buoy triplicate measurement example code:

```
//Prototype using all channels simultaneously takes 3 samples per time turned on

#include <Wire.h>
#include <SD.h>
#include <SPI.h>
```

```
#include "RTClib.h"

//PGA
//const int PGA = 0x00; // gain=1
//const int PGA = 0x01; // gain=2
const int PGA = 0x02; // gain==4
//const int PGA = 0x03; // gain=8

//Device addresses 1101
//Pair 1 Adr0=0; Adr1=float (001)
const int addr1 = 0x69;
const int chan1 = 4;

const int addr2 = 0x69;
const int chan2 = 3;

const int addr3 = 0x69;
const int chan3 = 2;

const int addr4 = 0x69;
const int chan4 = 1;

//Pair 6 Adr0=1; Adr1=0  (100)
const int addr5 = 0x6C;
const int chan5 = 4;

const int addr6 = 0x6C;
const int chan6 = 3;

const int addr7 = 0x6C;
const int chan7 = 2;

const int addr8 = 0x6C;
const int chan8 = 1;

//Pair 11 Adr0=0; Adr1=1 (110)
const int addr9 = 0x6A;
const int chan9 = 4;

const int addr10 = 0x6A;
const int chan10 = 3;

const int addr11 = 0x6A;
const int chan11 = 2;

const int addr12 = 0x6A;
const int chan12 = 1;

//Pair 16 Adr0=1; Adr1=1 (110)
const int addr13 = 0x6E;
const int chan13 = 4;

const int addr14 = 0x6E;
const int chan14 = 3;

const int addr15 = 0x6E;
const int chan15 = 2;

const int addr16 = 0x6E;
const int chan16 = 1;

//Link to RTC
RTC_PCF8523 rtc;
//The SD uses pin 10 on the Feather;
const int SDchipSelect = 10;

// the setup function runs once when you press reset or power the board
void setup() {
  // initialize digital pins 5,6,13 as outputs
  pinMode(5, OUTPUT); //Done pin for the timer
```

```
  pinMode(6, OUTPUT); //EN for the 5V regulator
  pinMode(13, OUTPUT); //light blinker

  digitalWrite(6, HIGH);  //Keep high to enable the 5V regulator
  digitalWrite(5, LOW);  //Start low; set to high when ready to shutdown timer

  Serial.begin(57600);
  Wire.begin();

 // Initialize the RTC:
  if (rtc.begin()) {
    Serial.println("RTC initialized.");
  } else
    Serial.println("RTC NOT initialized.");

  // Initialize the SD Card:
  if (SD.begin(SDchipSelect)) {
    Serial.println("SD card initialized.");
  } else
    Serial.println("SD card NOT initialized.");

 // Initialize the RTC:
  if (rtc.begin()) {
    Serial.println("RTC initialized.");
  } else
    Serial.println("RTC NOT initialized.");
}

// the loop function runs forever
void loop() {

  double volt[16];
  String timeStamp;
  String dataSample;
  DateTime now;

  // Open the output file for writing/appending
  File dataFile = SD.open("FC1.txt", FILE_WRITE);

  delay(1000);

  //take 3 samples
  for (int count=0; count < 3; count++) {

      delay(1000); //wait a second for the first/next sample

      // make a string for assembling the data to log:
      timeStamp = "";
      dataSample = "";
      now = rtc.now();

      timeStamp += now.year();
      timeStamp += "-" + addleadZero(now.month()) + "-";
      timeStamp += addleadZero(now.day()) + " ";
      timeStamp += addleadZero(now.hour()) + ":";;
      timeStamp += addleadZero(now.minute()) + ":";
      timeStamp += addleadZero(now.second());

      volt[0] = getVoltage(addr1,chan1);
      Serial.print("Volt_1 ");
      Serial.println(volt[0],3);
      volt[1] = getVoltage(addr2,chan2);
      Serial.print("Volt_2 ");
      Serial.println(volt[1],3);
      volt[2] = getVoltage(addr3,chan3);
      Serial.print("Volt_3 ");
      Serial.println(volt[2],3);
      volt[3] = getVoltage(addr4,chan4);
      Serial.print("Volt_4 ");
      Serial.println(volt[3],3);
```

```
volt[4] = getVoltage(addr5,chan5);
Serial.print("Volt_5 ");
Serial.println(volt[4],3);
volt[5] = getVoltage(addr6,chan6);
Serial.print("Volt_6 ");
Serial.println(volt[5],3);
volt[6] = getVoltage(addr7,chan7);
Serial.print("Volt_7 ");
Serial.println(volt[6],3);
volt[7] = getVoltage(addr8,chan8);
Serial.print("Volt_8 ");
Serial.println(volt[7],3);
volt[8] = getVoltage(addr9,chan9);
Serial.print("Volt_9 ");
Serial.println(volt[8],3);
volt[9] = getVoltage(addr10,chan10);
Serial.print("Volt_10 ");
Serial.println(volt[9],3);
volt[10] = getVoltage(addr11,chan11);
Serial.print("Volt_11 ");
Serial.println(volt[10],3);
volt[11] = getVoltage(addr12,chan12);
Serial.print("Volt_12 ");
Serial.println(volt[11],3);
volt[12] = getVoltage(addr13,chan13);
Serial.print("Volt_13 ");
Serial.println(volt[12],3);
volt[13] = getVoltage(addr14,chan14);
Serial.print("Volt_14 ");
Serial.println(volt[13],3);
volt[14] = getVoltage(addr15,chan15);
Serial.print("Volt_15 ");
Serial.println(volt[14],3);
volt[15] = getVoltage(addr16,chan16);
Serial.print("Volt_16 ");
Serial.println(volt[15],3);


//Assemble the data string
dataSample += timeStamp;
dataSample += ",";
dataSample += String(volt[0],3);
dataSample += ",";
dataSample += String(volt[1],3);
dataSample += ",";
dataSample += String(volt[2],3);
dataSample += ",";
dataSample += String(volt[3],3);
dataSample += ",";
dataSample += String(volt[4],3);
dataSample += ",";
dataSample += String(volt[5],3);
dataSample += ",";
dataSample += String(volt[6],3);
dataSample += ",";
dataSample += String(volt[7],3);
dataSample += ",";
dataSample += String(volt[8],3);
dataSample += ",";
dataSample += String(volt[9],3);
dataSample += ",";
dataSample += String(volt[10],3);
dataSample += ",";
dataSample += String(volt[11],3);
dataSample += ",";
dataSample += String(volt[12],3);
dataSample += ",";
dataSample += String(volt[13],3);
dataSample += ",";
dataSample += String(volt[14],3);
```

```
      dataSample += ",";
      dataSample += String(volt[15],3);

      Serial.println(dataSample);
      if (dataFile) {
        Serial.println("Writing to SD card");
        dataFile.println(dataSample);
        //dataFile.close(); close this outside the for loop instead
      } else {
        Serial.println("Error opening the SD file");
      }

  }//for loop

  dataFile.close();
  delay(200); //wait a bit for the SD to close the file

  digitalWrite(5, HIGH);  //Set to high when ready to shut down timer

}
double getVoltage(int address, int channel) {

  unsigned int data[2];
  int configByte = 0x80 + PGA;
  if (channel == 2) configByte = 0xA0 + PGA;
  if (channel == 3) configByte = 0xC0 + PGA;
  if (channel == 4) configByte = 0xE0 + PGA;

  Wire.beginTransmission(address);
  //Select configuration command
  Wire.write(configByte);
  Wire.endTransmission();
  delay(100);

  Wire.beginTransmission(address);
  //select data register
  Wire.write(0x00);
  Wire.endTransmission();
  delay(100);

  Wire.requestFrom(address,2);
  delay(100);
  if (Wire.available() == 2) {
      data[0] = Wire.read();
      data[1] = Wire.read();
  } else {
      data[0] = 0;
      data[1] = 0;
      //Serial.print(Wire.available());
      //Serial.println(" Data is not available");
  }

  int raw_adc = (data[0] & 0x0F) * 256 + data[1];
  if (raw_adc > 2047) {
     raw_adc -= 4096;
  }

  //Serial.print("RawADC ");
  //Serial.println(raw_adc);
  double delvolt = -1*raw_adc/1000.0;
  return (delvolt);

}
String addleadZero(int value) {
  String result = "0";
  if (value < 10) {
    result += value;
  }
  else {
    result = value;
```

```
  }
  return (result);
}
```
