# Supplementary material for: Cryptic cycling by electroactive bacterioplankton in Trout Bog Lake
Source: Appl Environ Microbiol. 2025 Jun 20;91(7):e01789-24. doi: 10.1128/aem.01789-24 (PMC12285243; doi:10.1128/aem.01789-24)
Supplement: Text S2 — Additional methods and text. [file aem.01789-24-s0002.pdf]

Supplementary Text S2: Additional methods and information

Additional Metagenomic Notes

Chlorobium sp. "3m_metabat_bin_57" (Tab. S1), referred to as GSB-B, blooms over the course of the summer just below the oxycline. Although it is uncertain as to whether Trout Bog *Chlorobium* spp. are actively metabolizing at night, we understand that during the day they require a source of reducing power, *i.e.* electron intake, to reduce CO_2 to biomass for anoxygenic photosynthesis. Regarding nocturnal metabolism, there exists some evidence that members of *Chlorobium* can ferment (Badalamenti *et al.*, 2014) and less evidence for respiration (Badalamenti *et al.*, 2013). The *Chlorobium* spp. in Trout Bog Lake have genes that suggest fermentation is possible, (*e.g.*, degrading glycogen stores), but also have the genes for nitric oxide reduction, oxygen reduction, and even 2–3 genes that match membrane proteins—FmnB+DmkB or FmnA+DmkAB+Ndh2 depending on the MAG—that catalyze electron transfer into the quinone pool in other organisms to selectively use particular electron acceptors (Unden and Bongaerts, 1997; Light *et al.*, 2018).

Sequencing resulted in MAGs similar to previous years (Olmsted *et al.*, 2022) with some notable exceptions. One major exception, at 0–1 m deep, most sequences (51% and 23%, respectively) mapped to a 225.5 kb single-contig MAG, 0m_metabat_bin_56, that is likely a virus (Fig. S10) in the order Caudoviricetes, based on the MVP pipeline (gitlab.com/ccoclet/mvp). Otherwise, the most common bacteria were in clades expected to respire oxygen and consume organic matter, such as Actinobacteria and *Novosphingobium*. However, one of the more abundant epilimnion-dwelling bacteria was classified within *Steroidobacteraceae*, and it harbored both oxiEET and redEET genes (Tab. 1).

Metagenomics suggests additional EET-capable bacteria may be involved in cryptic cycling of forms of sulfur and nitrogen around 4m deep, particularly members of the following taxa. *Ferrovaceae* may be capable of oxiEET, redEET, methanol oxidation, carbon fixation, sulfide oxidation, and thiosulfate redox. *Terracidiphilus* sp. seems capable of oxiEET, redEET, hydrogen metabolism, sulfur(ide) oxidation, nitrate(ite) reduction, and acetogenic fermentation. *Magnetospirillaceae* appear capable of oxiEET, redEET, sulfide oxidation, and thiosulfate redox, methanol oxidation, and fermentation. Some of these organisms might also use oxiEET for their electron source while respiring fermentation products for an energy source or use oxiEET for chemoautotrophy.

Additional Electrical Results

For analysis, we separated data from the time series into four periods: through the first full day after deployment (28 h), and the first, middle, and last thirds of the 17-day deployment. As biofilms grew throughout these four periods, we observed significant correlations between environmental data and the diel component of electrical current, *i.e.* the amperage minus the average of amperages ranging from 12 h before to 12 h after. Hourly averages of the diel component over the course of these four periods resulted in obvious waveform patterns (Fig. S1). For example, Channel 11 (Fig. S1C11) was connected such that electrons flowed from the depth range most enriched with *Chlorobium* spp. (2.5±0.5 m), cooccurring with *Geothrix* sp., towards a cathode at 10cm. Channel 11 displayed a waveform that significantly negatively correlated to light intensity ($r = -.43, -.36, -.45$) until the last third ($r = -.07$) of the dataset when its trend in current increased dramatically (Fig. S7) and changed the waveform's diel pattern. The diel component of current for Channel 11 also significantly positively correlated with dissolved

oxygen at 20 cm (r = .75, .41, .28, .38) and temperature at 20 cm (r = .55, .10, .22, .48) throughout the dataset.

Electron flow from anode to cathode for all channels with an electrode in the *Chlorobium*-enriched depth range, 2.5±0.5 m, (Fig. S1C1&2&6&7–11&14&16) was significantly negatively correlated with surface light for at least one of the four periods while this was the case for only half of channels with connections from 4.75±1.25 m to 10 cm (Fig. S1C2&4&12&15). Also, the average time that channels' current flow away from 2.5±0.5 m significantly negatively correlated to light was 44% higher than for channels connected from 4.75±1.25 m to 10 cm (Fig. S1A&B).

Some more explorative channels were constructed to test for potential phenomena that might impact the observed electrical currents. For example, certain channels had one or both electrodes covered in microporous (~12−16 nm) calcium alginate mesh to limit non-mediated EET (Nevin and Lovley, 2000). These channels showed different waveform patterns and correlations compared to naked-electrode channels of similar depths, including overall delayed diel waveforms and/or peaks rather than troughs (Fig. S1). To test more exploratively for non-*Chlorobium* phototroph-related waveform patterns, we placed select cathodes in the oxycline at 70 cm. As light and temperature increased, fewer electrons were accepted from these 70 cm-deep cathodes (Fig. S1C5&13).

Metagenomics Assembly

Metagenomic library preparation and Illumina DNA sequencing (Pooled 150PE NovaSeq S4) occurred as indicated (Supp. Tab. 2) through the Research and Technology Support Facility Genomics Core at Michigan State University (400 M read pairs across samples) or California Institute for Quantitative Biosciences (10 Gb per sample). To process the metagenomic data,

sequence reads were quality-filtered using fastp 0.20.0 (Chen *et al.*, 2018) and assembled into scaffolds using metaSPAdes v3.13.1 (Bankevich *et al.*, 2012). Scaffolds were quality-filtered using anvi'o v6.1 (Eren *et al.*, 2021). Reads were mapped to scaffolds using bowtie2 (Langmead and Salzberg, 2012). MetaBAT2 v2.12.1 (Kang *et al.*, 2015) was used to bin scaffolds into MAGs. Taxonomy was assigned using GTDB-tk v0.3.2 (Chaumeil *et al.*, 2020). MAG completeness and contamination were assessed using CheckM v1.1.2 (Parks *et al.*, 2015).

Chemical Analysis

Water samples for sulfide analysis were stabilized upon collection by adding zinc acetate to a final concentration of 1% and analyzed by photometry at 667 nm using the Cline method (Cline, 1969; Reese *et al.*, 2011). Metals samples in 2017 were stabilized by adding nitric acid to a final concentration of 2% and analyzed by inductively coupled plasma mass spectrometry (Thermo Fisher Scientific, iCAP-RQ) (Peterson *et al.*, 2020). On September 5th, 2021, iron samples were stabilized by adding nitric acid to a concentration of 2%. These samples were digested with final concentrations of 1.54% nitric acid, 4% hydrochloric acid, and 3.69% hydrogen peroxide and analyzed by inductively coupled plasma optical emission spectrometry (ICP-OES, Agilent 5110 VDV). Matrix agreement between ICP-OES samples and standards was evaluated with a subset of ferrous chloride matrix spike solutions, and recovery was within 100 ± 10% for each test. Signal intensity was corrected for drift using an internal yttrium standard, and error for iron concentration in each sample was set as the sum of relative standard deviation of replicate measurements and relative standard error on the external calibration curve (Fig. S4A). Other ICP-OES elemental data was collected semi-quantitatively simultaneously with iron data collection. Instrument response for other elements present in the measured material is compared against a factory calibration, eliminating the need for calibration at the expense of certainty. The

resulting data were considered with the largest uncertainty (±35.53%) from the semi-quantitative data of the iron calibration solutions.

Graphical and Statistical Analysis

Diel profile graphs, taxonomic bar charts, electrical current line graphs, and heatmaps, were generated using ggplot2 (Wickham, 2009) package from R (R Core Team, 2014). Pearson correlations, time-lagged cross correlations (also Pearson correlation), and multivariate linear regressions were also generated using R. R packages used include patchwork (Pedersen, 2020), and dplyr (Wickham *et al.*, 2020), zoo (Zeileis and Grothendieck, 2005), reshape2 (Wickham, 2007), and lubridate (Grolemund and Wickham, 2011), and R code used is publicly available (github.com/McMahonLab/Scripts/rawRcode). Significance of ORP shifts were analyzed by Welch Two Sample T-tests in R. Seasonal heatmap interpolation graphs were generated using Matlab v9.12.0 (The MathWorks Inc., 2010).

ProDSS ORP Probe Testing

Laboratory tests were conducted with the ProDSS sonde to examine the response of the ORP probe to changes in pH and dissolved O_2 analogous to those observed within the upper few meters of Trout Bog Lake. The experiments employed solutions buffered to pH values between 3.5 and 4.5 with 0.35 mM potassium hydrogen phthalate, which produced specific conductivity values like those in Trout Bog waters. Eliot Soil IHSS humic acid (20 mg/L) and $FeCl_3$-$6H_2O$ (10 μM) were added to simulate redox-active compounds present above the redoxcline in the bog. Additionally, solutions of humic acids, iron, or the phthalate alone and in combination were subjected to the same experiments. In some experiments, solutions were bubbled with O_2-free N_2 until dissolved O_2 decreased past the detection limit of the ProDSS. No reducing agents were added to the N_2-bubbled solutions, such that both humic acid and Fe were assumed to remain in

their oxidized form during the one-hour deoxygenation period. We also conducted experiments by changing pH using HCl or NaOH. These experiments revealed that anoxia decreases ORP, but the magnitude change was inconsistent between trials. Also, decreasing pH increases ORP ~50 mV per unit pH. By our calculations, the increase in conductivity at ~1.5 m and some of the increase in ORP at ~1.5 m and can be explained by the pH decrease at that depth. In the context of seasonal data, at most 10% of seasonal ORP increase may be explained by pH decline, some of which would presumably be caused by the production and hydrolysis of Fe^{3+} generated during photoelectrotrophic activity. However, the increases in the weekly fluctuations of ORP 2 m and below did not visually align with weekly decreases in pH, nor the dates of calibration, nor variations in oxygen, and thus remain unexplained by the abiotic factors we measured. Also, field measurements of ORP and pH variably correlated (Pearson correlation as listed next), suggesting there may have been other factors that affected ORP more than varying pH and, considering the stable oxycline, more than variations in oxygen. Correlations of pH and ORP data throughout the whole bog: 2018 ($R = -.317$, $p = 4.8e{-60}$), 2019 ($R = +.140$, $p = 8.8e{-18}$), 2021 ($R = -.244$, $p = 6.8e{-71}$). Between 1.5 to 2.5m: 2018 ($R = -.701$, $p = 2.3e{-83}$), 2019 ($R = +.13$, $p = 3.5e{-4}$), 2021 ($R = +.109$, $p = 9.1e{-4}$).

REFERENCES

Badalamenti, J.P., Torres, C.I., and Krajmalnik-brown, R. (2014) Coupling Dark Metabolism to Electricity Generation Using Photosynthetic Cocultures. *Biotechnol Bioeng* **111**: 223–231. https://doi.org/10.1002/bit.25011.

Badalamenti, J.P., Torres, I., and Krajmalnik-brown, R. (2013) Light-Responsive Current Generation by Phototrophically Enriched Anode Biofilms Dominated by Green Sulfur Bacteria. *Biotechnol Bioeng* **110**: 1020–1027. https://doi.org/10.1002/bit.24779.

Bankevich, A., Nurk, S., Antipov, D., Gurevich, A.A., Dvorkin, M., Kulikov, A.S., et al. (2012) SPAdes: A New Genome Assembly Algorithm and Its Applications to Single-Cell Sequencing. *J Comput Biol* **19**: 455–477. https://doi.org/10.1089/cmb.2012.0021.

Chaumeil, P.-A., Mussig, A.J., Hugenholtz, P., and Parks, D.H. (2020) GTDB-Tk: a toolkit to classify genomes with the Genome Taxonomy Database. *Bioinformatics* **36**: 1925–1927.

https://doi.org/10.1093/bioinformatics/btz848.
Chen, S., Zhou, Y., Chen, Y., and Gu, J. (2018) Fastp: an ultra-fast all-in-one FASTQ preprocessor. *Bioinformatics* **34**: i884–i890. https://doi.org/10.1093/bioinformatics/bty560.
Cline, J.D. (1969) Spectrophotometric determination of sulfide in natural waters. *Limnol Oceanogr* **14**: 454–458. https://doi.org/10.4319/lo.1969.14.3.0454.
Eren, A.M., Kiefl, E., Shaiber, A., Veseli, I., Miller, S.E., Schechter, M.S., et al. (2021) Community-led, integrated, reproducible multi-omics with anvi'o. *Nat Microbiol* **6**: 3–6. https://doi.org/10.1038/s41564-020-00834-3.
Grolemund, G. and Wickham, H. (2011) Dates and Times Made Easy with lubridate. *J Stat Softw* **40**: 1–25.
Kang, D.D., Froula, J., Egan, R., and Wang, Z. (2015) MetaBAT, an efficient tool for accurately reconstructing single genomes from complex microbial communities. *PeerJ* **3**: e1165. https://doi.org/10.7717/peerj.1165.
Langmead, B. and Salzberg, S.L. (2012) Fast gapped-read alignment with Bowtie 2. *Nat Methods* **9**: 357–359. https://doi.org/10.1038/nmeth.1923.
Light, S.H., Su, L., Rivera-Lugo, R., Cornejo, J.A., Louie, A., Iavarone, A.T., et al. (2018) A flavin-based extracellular electron transfer mechanism in diverse Gram-positive bacteria. *Nature* **562**: 140–144. https://doi.org/10.1038/s41586-018-0498-z.
Nevin, K.P. and Lovley, D.R. (2000) Lack of Production of Electron-Shuttling Compounds or Solubilization of Fe (III) during Reduction of Insoluble Fe (III) Oxide by Geobacter metallireducens. **66**: 2248–2251.
Olmsted, C.N., Ort, R., Tran, P.Q., McDaniel, E.A., Roden, E.E., Bond, D.R., et al. (2022) Environmental predictors of electroactive bacterioplankton in small boreal lakes. *Environ Microbiol* **25**: 705–720. https://doi.org/10.1111/1462-2920.16314.
Parks, D.H., Imelfort, M., Skennerton, C.T., Hugenholtz, P., and Tyson, G.W. (2015) CheckM: assessing the quality of microbial genomes recovered from isolates, single cells, and metagenomes. *Genome Res* **25**: 1043–1055. https://doi.org/10.1101/gr.186072.114.
Pedersen, T.L. (2020) patchwork: The Composer of Plots. R package version 1.1.1.
Peterson, B.D., McDaniel, E.A., Schmidt, A.G., Lepak, R.F., Janssen, S.E., Tran, P.Q., et al. (2020) Mercury Methylation Genes Identified across Diverse Anaerobic Microbial Guilds in a Eutrophic Sulfate-Enriched Lake. *Environ Sci Technol* **54**: 15840–15851. https://doi.org/10.1021/acs.est.0c05435.
R Core Team (2014) R: A language and environment for statistical computing. R Foundation for Statistical Computing, Vienna, Austria. URL http://www.R-project.org/.
Reese, B.K., Finneran, D.W., Mills, H.J., Zhu, M.-X., and Morse, J.W. (2011) Examination and Refinement of the Determination of Aqueous Hydrogen Sulfide by the Methylene Blue Method. *Aquat Geochemistry* **17**: 567–582. https://doi.org/10.1007/s10498-011-9128-1.
The MathWorks Inc. (2010) MATLAB.
Unden, G. and Bongaerts, J. (1997) Alternative respiratory pathways of Escherichia coli: energetics and transcriptional regulation in response to electron acceptors. *Biochim Biophys Acta - Bioenerg* **1320**: 217–234. https://doi.org/10.1016/S0005-2728(97)00034-0.
Wickham, H. (2009) ggplot2, New York, NY: Springer New York https://doi.org/10.1007/978-0-387-98141-3.
Wickham, H. (2007) Reshaping Data with the reshape Package. *J Stat Softw*.

Wickham, H., François, R., Henry, L., and Müller, K. (2020) dplyr: A Grammar of Data Manipulation.R package version 1.0.2.

Zeileis, A. and Grothendieck, G. (2005) zoo: S3 Infrastructure for Regular and Irregular Time Series. *J Stat Softw* **14**: 1–27. https://doi.org/doi:10.18637/jss.v014.i06.
