# Supplementary material for: Cryptic cycling by electroactive bacterioplankton in Trout Bog Lake
Source: Appl Environ Microbiol. 2025 Jun 20;91(7):e01789-24. doi: 10.1128/aem.01789-24 (PMC12285243; doi:10.1128/aem.01789-24)
Supplement: Supplemental figures — Figures S0 to S10. [file aem.01789-24-s0003.pdf]

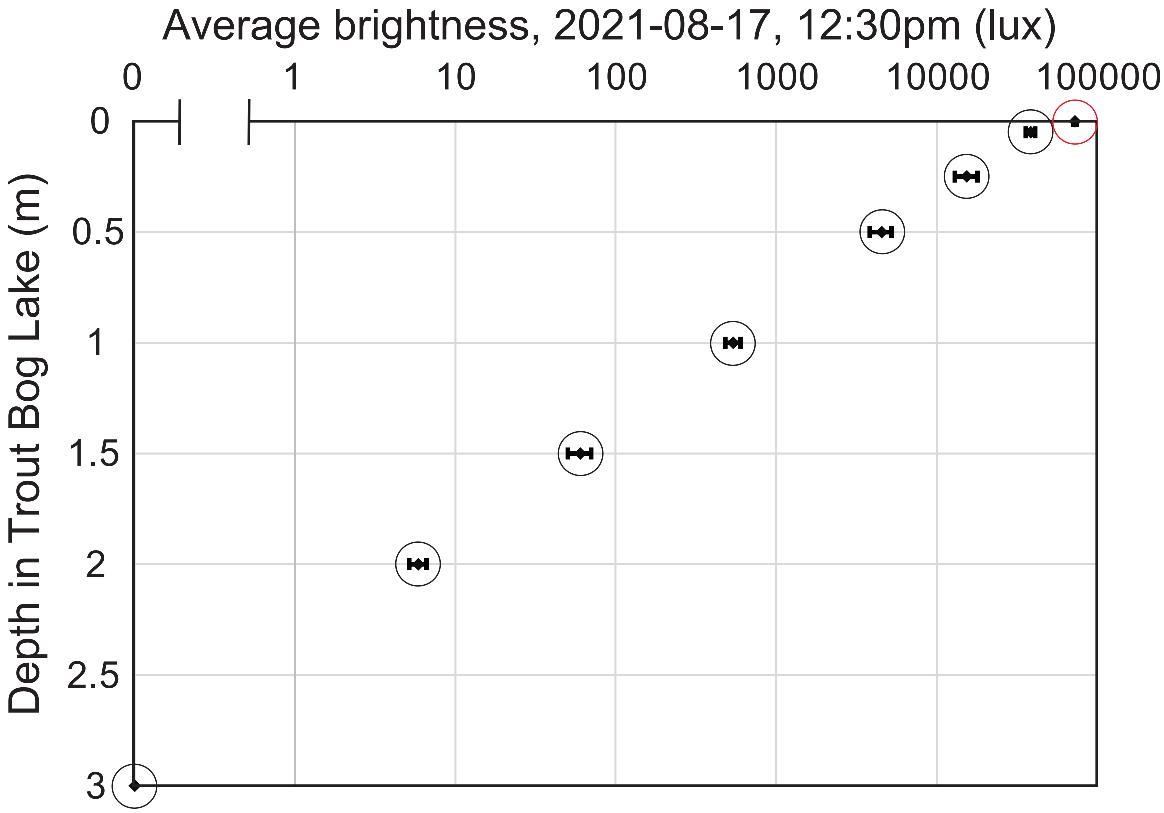

Supplementary Figure S0. Vertical brightness profile of Trout Bog Lake. A HOBO Onset MX2202 was lowered at the end of an L-shaped PVC pipe and recorded at each depth for at least 100 s, measuring brightness every 5 s. Error bars represent standard deviation (N~20). The red circled value was recorded above water. The black circled values were recorded when submerged.

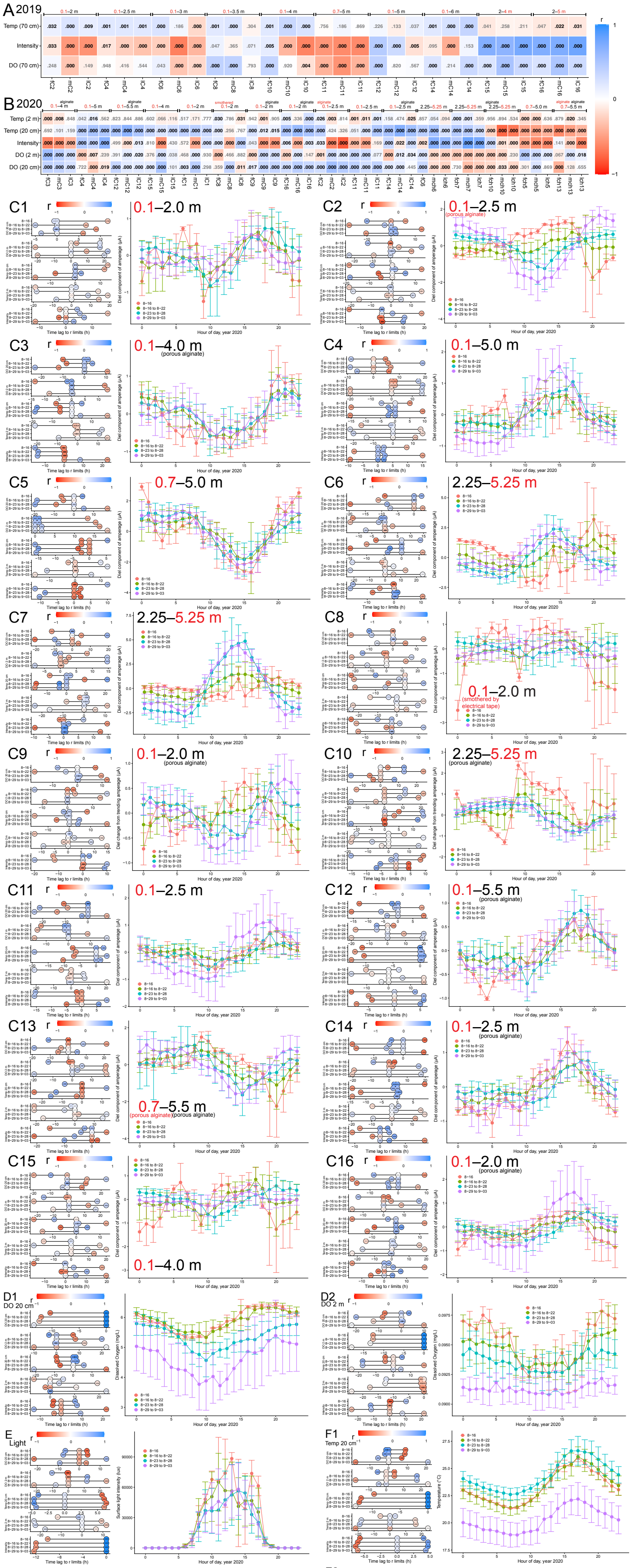

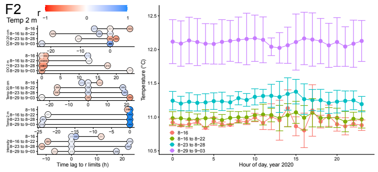

Supplementary Figure S1. Correlation heatmaps between (A) 2019 and (B) 2020 environmental measurements and circadian components of Microghost Buoy electrical measurements, and (C, right) diel patterns and (C, left) time-lag cross correlation analyses of 2020 buoy deployment including (C1–16) electrical to environmental comparisons and (D–F) environmental to environmental comparisons. Electrical measurements were between 16 pairs of electrodes at the indicated depths, each connected and measured across a 4.75 Ω resistor. Each electrode pair or "channel" corresponds to the figure numbers C1 to C16. Frequency decomposition was performed using a period of 24 h, and the resulting trend component was subtracted from amperage to generate the diel components. Time-lag analysis dot color and heatmap rectangle color represents Pearson correlation coefficient, r, calculated using the diel component of electrical current values. Unadjusted p-values listed as text inside the dot or rectangle are out of N=83, N=417, N=418, and N=417 available corelates respectively for the first day, first third (f), middle third (m), and last third (l) of the datasets. By "first day" we mean up to the first full day of deployment, including the afternoon of deployment plus 12:00 am to 12:00 am. Black instead of grey text indicates a significance of p<.05 when adjusted by the Benjamini-Hochberg method. Overall electron flow direction is towards the cathode, indicated by coloring the cathodic electrode's depth red. Positive r values relate to increased electron flow except when current was overall negative. Error bars are standard deviation, N~18. Statistical values were generated with the program R. Environmental values including dissolved oxygen (DO), Temperature (Temp) were measured at 2 m, 20 cm, or 70 cm deep as indicated using a separate buoy roughly 10 m away while light intensity was logged by a HOBO device on top of the Microghost Buoy. Positive time lag for the factor being compared indicates that the maximum or minimum r limit occurs when the values of the subject factor are lagged by that many hours, and all r values without any time lag are displayed as well.

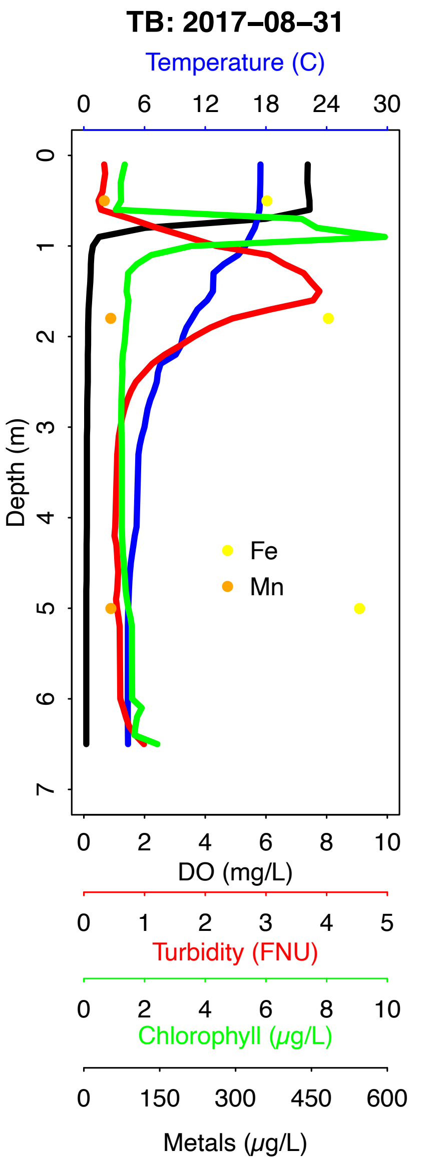

Supplementary Figure S2. Vertical profiles in 2017. Turbidity, dissolved oxygen (DO), chlorophyll, and temperature were recorded by slowly dropping an EXO1 sonde. Metals were sampled by peristaltic pump at indicated depths, stabilized by adding nitric acid to a final concentration of 2%, and later analyzed inductively coupled plasma mass spectrometry (ICP-MS).

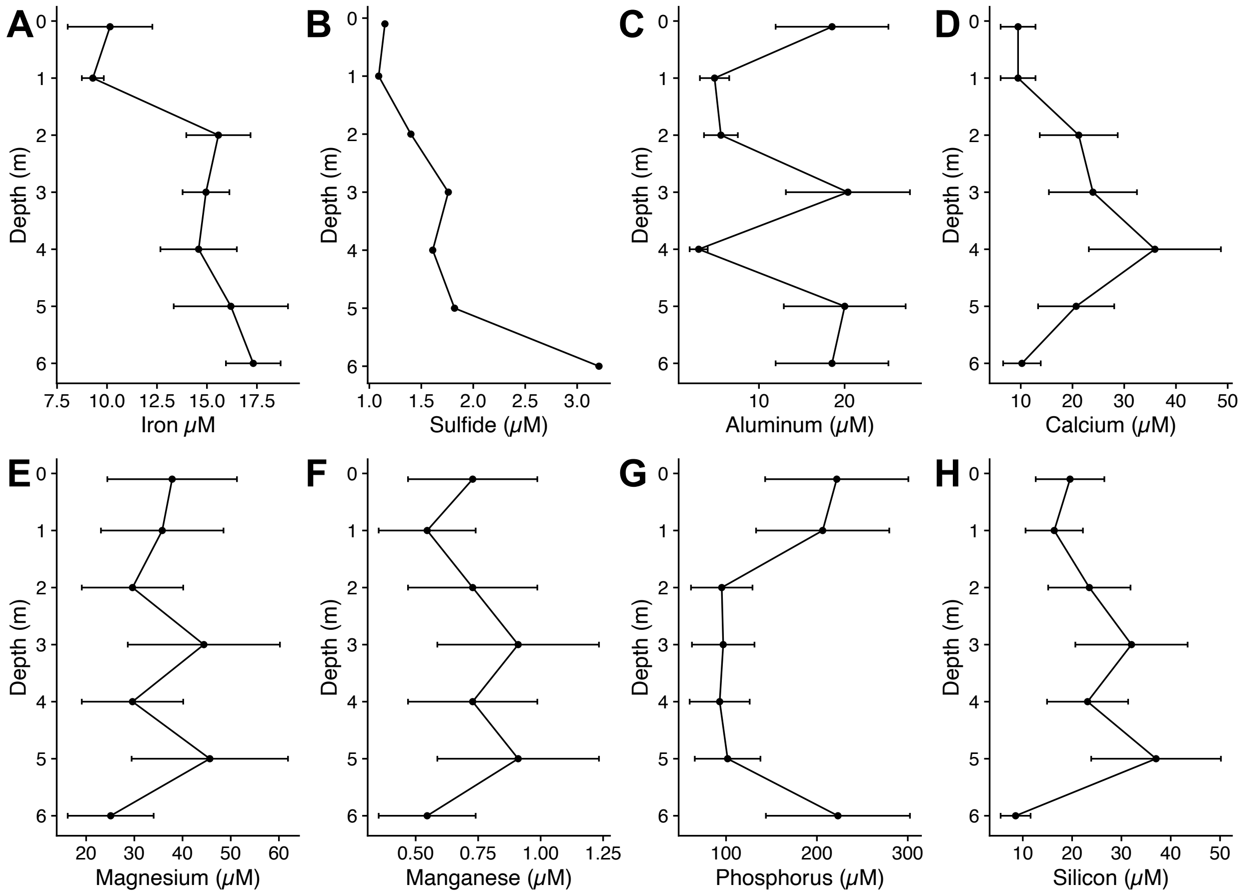

Supplementary Figure S4. Iron (A), sulfide (B), and other (C–H) elemental concentration profiles from September 5th, 2021. Sulfide samples were collected and analyzed according to the Cline method (Cline, 1969). Quantitative iron (error bars: standard deviation of replicate measurements) as well as relative concentrations of other elements (error bars: uncertainty of ± 35.530%) were obtained via inductively coupled plasma optical emission spectrometry (Agilent 5110) after peroxide-acid digestion.

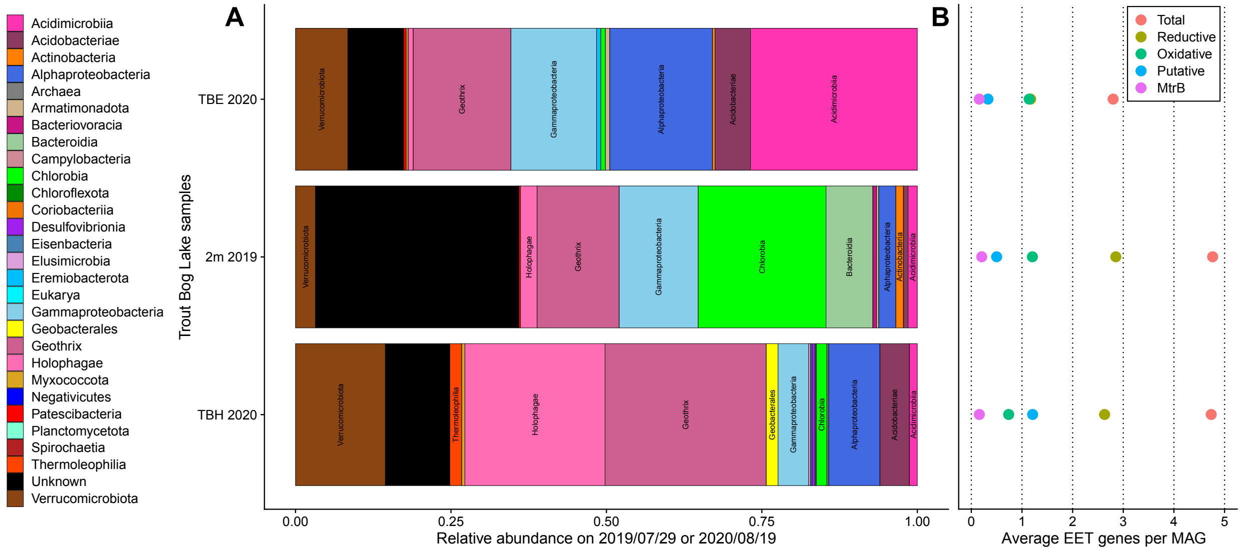

Supplementary Figure S5. Relative abundances of mOTUs (A) and Average EET gene counts per MAG (B) from a 2 m-deep 2019 sample and 2020 samples of integrated epilimnion and hypolimnion water. Integrated epilimnion water (TBE) was from 0 m to 2 m deep while hypolimnion (TBH) water was from 2 m to 7 m deep. Integration was achieved by peristaltically pumping from a tube dropped at a constant rate through the sample ranges. Samples were filtered through 0.2 µm to collect bacteria. Relative abundance represents the average metagenomic read mapping for all mOTUs in the given clade, and displayed subclades were not double counted in higher clades. Average EET gene counts are derived from sample-specific MAGs, not mOTUs.

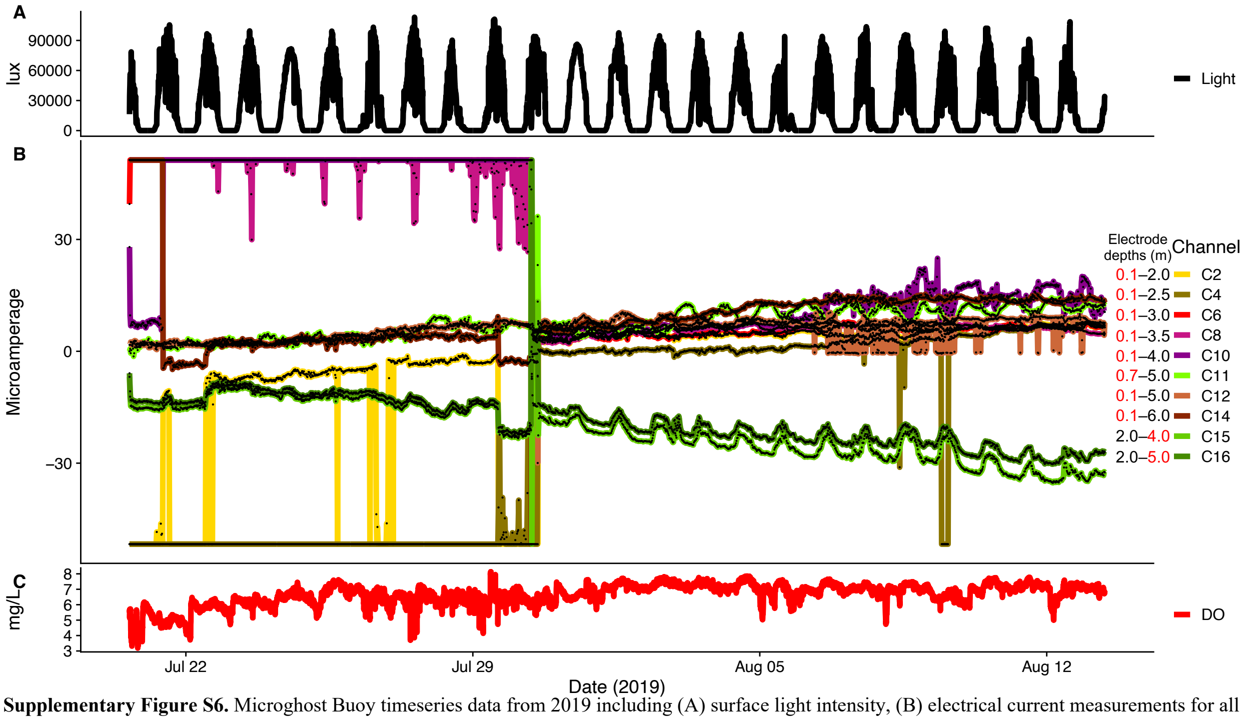

Supplementary Figure S6. Microghost Buoy timeseries data from 2019 including (A) surface light intensity, (B) electrical current measurements for all channels, (C) dissolved oxygen (DO) measured at 70 cm deep. All electrodes were plain carbon cloth connected by insulated copper wire, but some were not included due to an ill-conceived design. Depth (m) of cathodes (red text) and anodes (black text) is as indicated. The electrodes were initially loosely organized, causing frequent circuit shorts due to channel-on-channel contact until July 31st when we took care to secure the wires in place, eliminating most of the shorting.

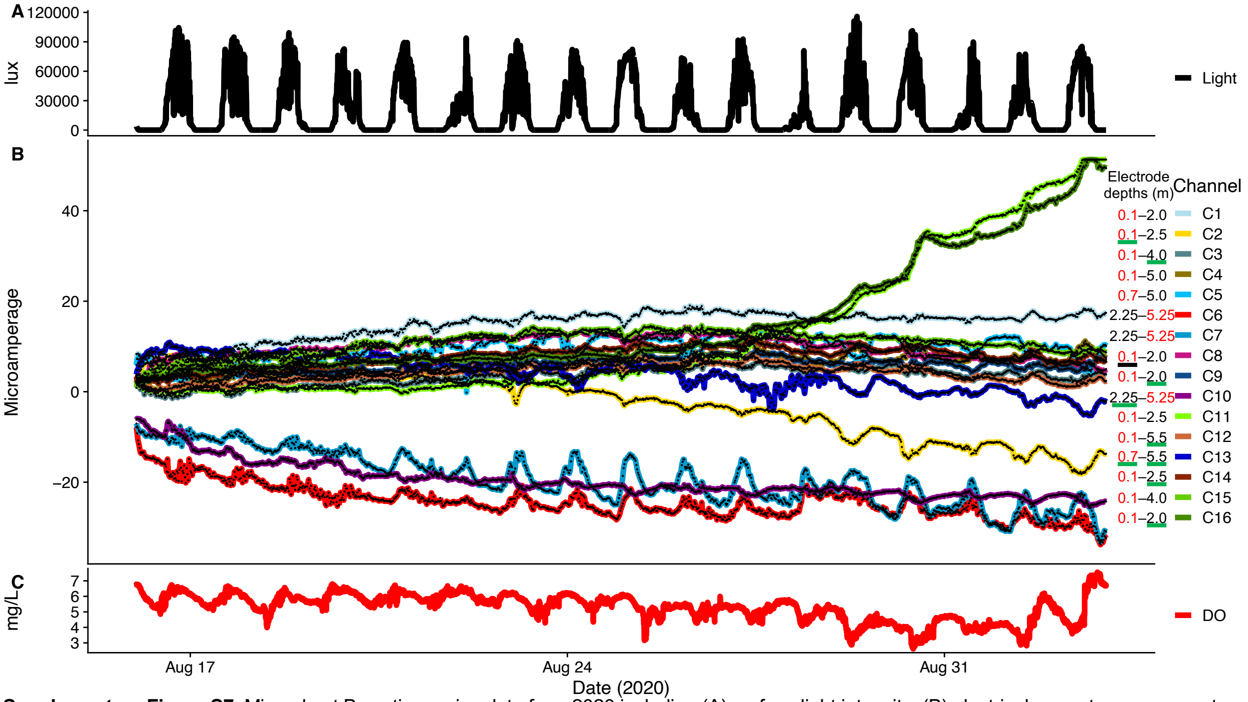

Supplementary Figure S7. Microghost Buoy timeseries data from 2020 including (A) surface light intensity, (B) electrical current measurements for all channels, (C) dissolved oxygen (DO) measured at 20 cm deep. Depth (m) of cathodes (red text) and anodes (black text) is as indicated. All electrodes were plain carbon cloth connected by insulated copper wire. Some electrodes were covered in microporous calcium alginate with an estimated pore size of 12–16 nm (green underline). One cathode was smothered in black electrical tape (black underline).

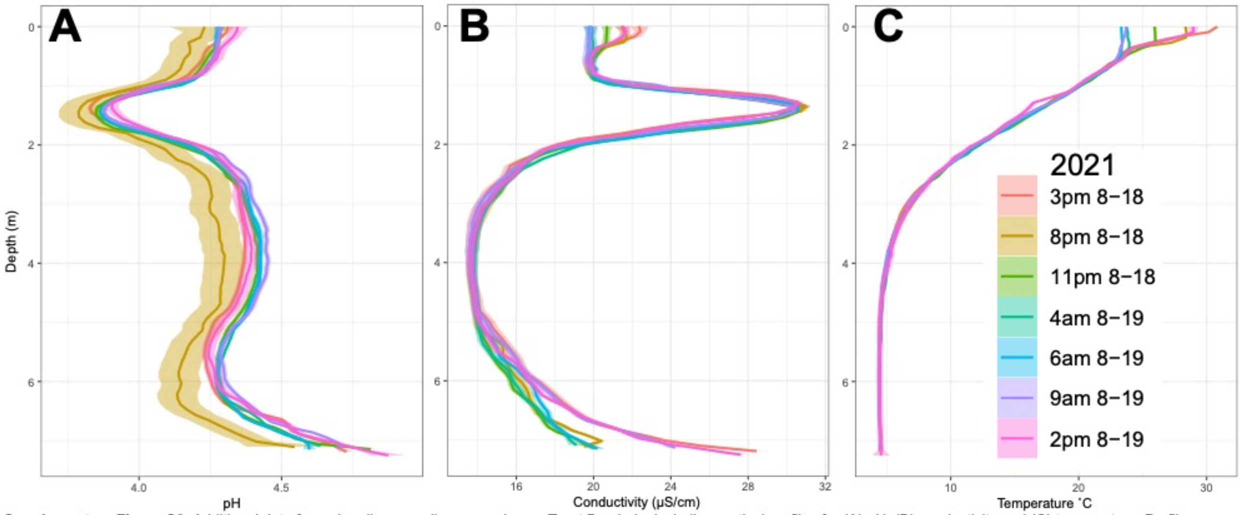

Supplementary Figure S8. Additional data from circadian sampling campaign on Trout Bog Lake including vertical profiles for (A) pH, (B) conductivity and (C) temperature. Profiles were collected by slowly lowering a ProDSS sonde. Each timepoint is an average of three profiles whose data are 20 min apart because each profile took 15 min to record. Profiles were collected between August 17th, 2021, and August 19th, 2021.

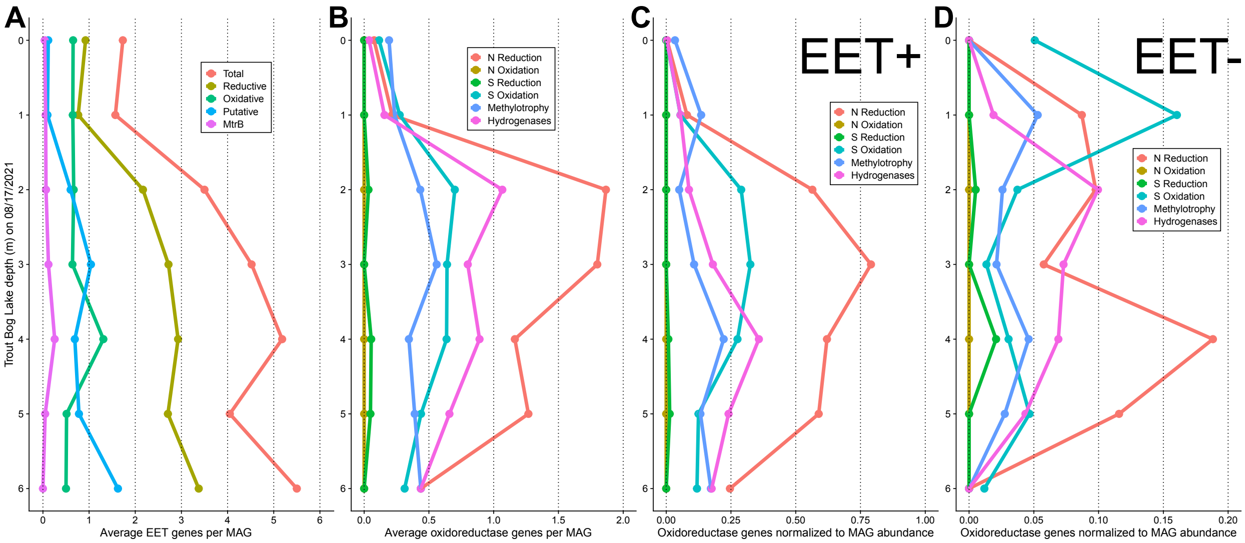

Supplementary Figure S9. Oxidoreductase values by depth in Trout Bog Lake including (A) average EET gene counts per MAG, (B) average other oxidoreductase counts per MAG, and oxidoreductase genes normalized to MAG abundance of MAGs (C) with identified EET genes and (D) without. Values are derived from sample-specific MAGs, not mOTUs. Average gene counts are total sample counts of the given gene divided by the number of MAGs in that sample. Abundance-normalized gene counts were calculated by multiplying each MAG's number of EET genes by the relative abundance of that MAG and adding all such values together per sample.

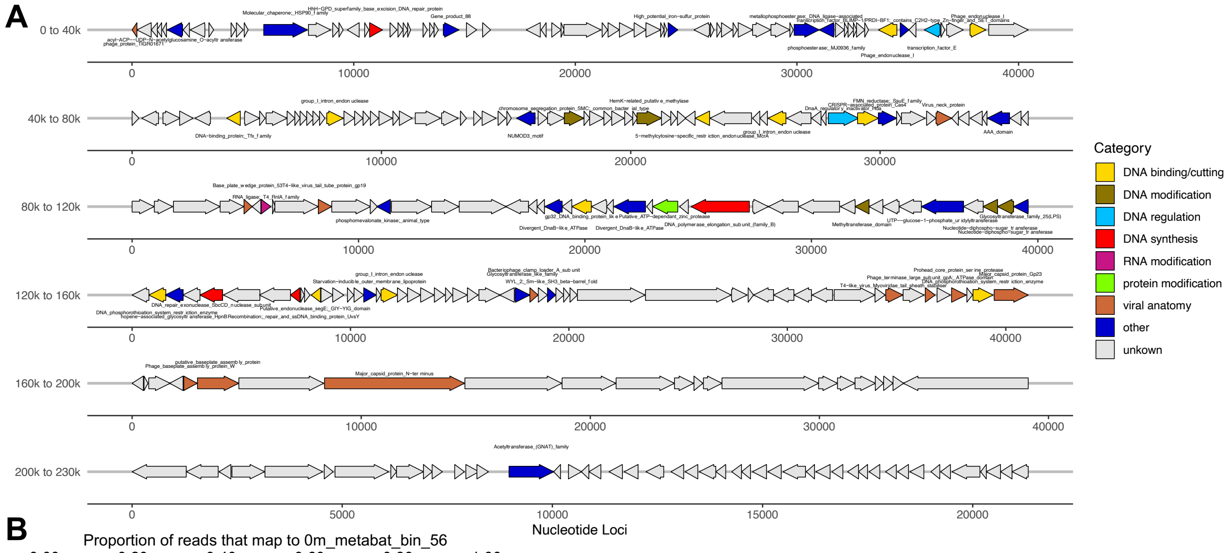

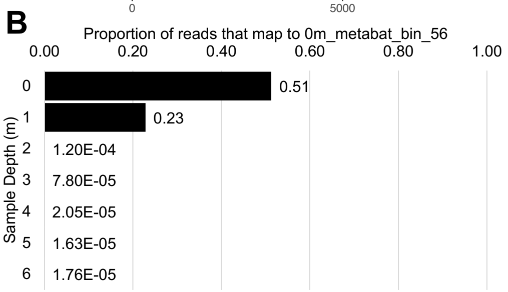

Figure S10. Genetic map (A) and relative abundance (B) of 0m_metabat_bin_56. Only one contiguous nucleotide sequence represents 0m_metabat_bin_56, but it is split here into rows. Rows are scaled differently. Centered upon colored arrows, each gene's geNomad (github.com/apcamargo/genomad) annotation is listed above (forward) or below (reverse) depending on the DNA strand. Graph generated using the gggenes R package. To calculate relative abundance, each given sample's reads were mapped against MAGs from the 0 m sample.
