# Supplementary material for: Cryptic cycling by electroactive bacterioplankton in Trout Bog Lake
Source: Appl Environ Microbiol. 2025 Jun 20;91(7):e01789-24. doi: 10.1128/aem.01789-24 (PMC12285243; doi:10.1128/aem.01789-24)
Supplement: Supplemental legends — Legends for Tables S1 to S5. [file aem.01789-24-s0004.pdf]

SUPPLEMENTARY TABLE LEGENDS

Supplementary Table S1. Metagenome assembled genome (MAG)-specific information. This dataset lists all MAGs for all included samples and to which mOTU each belongs. No MAGs were removed, even poor-quality ones, but CheckM completeness and contamination values are given. mOTURelAbund is the relative abundance of the listed mOTU based on read mapping of the given sample to all mOTUs while RelAbund is the relative abundance based on read mapping to MAGs derived from that sample. TotEET, RedEET, OxiEET, PutEET, MtrB are values generated by the FEET pipeline on every MAG, respectively total, reductive, oxidative, putative, and MtrB EET genes. MtrB is a porin that can be involved in either OxiEET or RedEET. The values to the right of these are for each listed mOTU and are from the modified METABOLIC software in which we added hidden Markov models for EET genes from the FEET pipeline, but these may not always be counted as an EET gene by FEET rules. MAGs available at https://osf.io/kmj2s/.

Supplementary Table S2. Sample-specific information. Sample DNA was sequenced by cited efforts, RTSF Genomics Core at Michigan State University (MSU), or California Institute for Quantitative Biosciences (QB3). All reads available for open access under the NCBI Bioproject ID PRJNA1018295.

Supplementary Table S3. Electroactive chemical species in Trout Bog Lake. Data are averages from all available samples during 2016–2021.

Supplementary Table S4. Multivariate linear regression analysis summary with diel component of current as the dependent variable. Best predicting individual independent variables are listed first and followed by consecutive addition of the next best predicting variables.

Supplementary Table S5. A key of abbreviations.
